# Beyond symmetric self-assembly and effective molarity: unlocking functional enzyme mimics with robust organic cages

**DOI:** 10.3762/bjoc.21.30

**Published:** 2025-02-24

**Authors:** Keith G Andrews

**Affiliations:** 1 Department of Chemistry, Durham University, Lower Mount Joy, South Rd, Durham, DH1 3LE, UK

**Keywords:** cavity confinement catalysis, enzyme mimicry, robust organic cages, self-assembly, supramolecular catalysis

## Abstract

The bespoke environments in enzyme active sites can selectively accelerate chemical reactions by as much as 10^19^. Macromolecular and supramolecular chemists have been inspired to understand and mimic these accelerations and selectivities for applications in catalysis for sustainable synthesis. Over the past 60+ years, mimicry strategies have evolved with changing interests, understanding, and synthetic advances but, ubiquitously, research has focused on use of a molecular “cavity”. The activities of different cavities vary with the subset of features available to a particular cavity type. Unsurprisingly, without synthetic access to mimics able to encompass more/all of the functional features of enzyme active sites, examples of cavity-catalyzed processes demonstrating enzyme-like rate accelerations remain rare. This perspective will briefly highlight some of the key advances in traditional cavity catalysis, by cavity type, in order to contextualize the recent development of robust organic cage catalysts, which can exploit stability, functionality, and reduced symmetry to enable promising catalytic modes.

## Introduction

I frequently introduce my research on organic cage enzyme mimics with the following observation. For hundreds of years, chemists have made use of selectively reactive chemicals by handling them, individually, in unreactive bottles. Meanwhile, Nature has learned to convert mixtures of unreactive chemicals by handling them in selectively reactive bottles (enzymes). Only the latter approach offers the efficiency, rate-accelerations (10^19^) [1], tolerance of contaminants, and selectivity associated with the power of enzymes. It is for this reason that I have sought to introduce supramolecular approaches into my organocatalysis [2].

**Figure 1:**
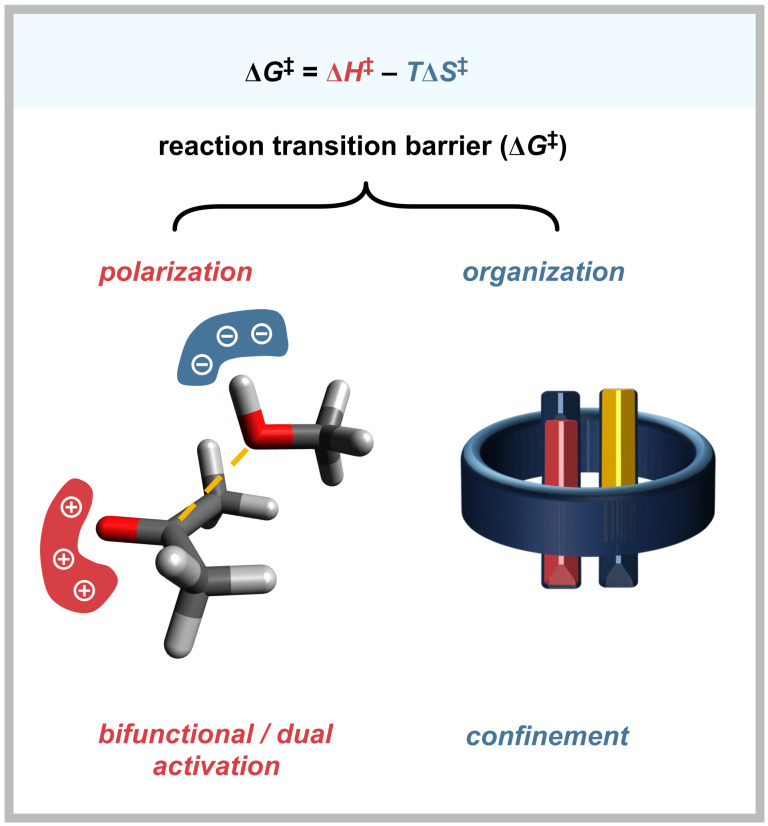
Catalytic rate enhancements from a reduction in the Gibbs free energy transition barrier can be framed in terms of contributions from *polarization* and *organization* by the catalyst/system.

From thermodynamics, there are two components to catalysis: *organization* (entropic) and *polarization* (enthalpic) (Figure 1). *Organization,* the control of the position(s) and orientation(s) of reacting molecules, has been achieved historically in supramolecular chemistry using “pre-organized” catalyst designs (vide infra). More recently, *organization* has been mooted by List as a unifying concept across many fields of selective catalysis under the term *confinement* [3], a term borrowed from heterogeneous catalysis. *Polarization* can be understood as the catalyst providing an electrostatic environment that works to stabilize electron redistribution. Since all reactions redistribute electrons, and any charge generation requires that an equal and opposite charge be generated elsewhere, the unifying concept for *polarization* is that of “cooperative [4] bifunctionality”: providing opposing functionalities able to stabilize opposite charges: *dual activation* (e.g., the simultaneous activation of nucleophile and electrophile) [5–12].

Crucially, enzymes *organize* their *polarization* – they achieve both components in tandem. Supramolecular chemists have made significant advances in cavity catalysis [13–24] – albeit slowly [25] – but have predominantly achieved *organization* and *polarization* independently [26]. As we shall see, there has been a bias towards *organization* (controlling reagent distributions), often leaving any catalysis to chance. This even appears to be a strategy: *“the search for supramolecular reactors that contain no catalytically active sites but can promote chemical transformations has received significant attention, but it remains a synthetic challenge* [27]*.”* The vague term “confinement” is sometimes used as a catch-all explanation for the property changes that arise within a cavity environment, often in the context of zeolites [28–32]. Undoubtedly property changes can occur in cavities, but those properties not covered by *organization* must be explicable: I would argue (for typical ground-state reactions) they are covered by the concept of *polarization*.

Modern descriptions of enzyme active sites speak of *organized polarization* rather than “confinement,” specifically positing that oriented electric fields rule transition-state stabilization for many reactions [33–35]. These fields stabilize charge redistribution during a reaction, usually at a small locus of each substrate molecule. It follows, therefore, that for redistribution penalties to be lessened, the equal and opposite stabilization must be granted to that same space. This is the basis of bifunctional/dual activation, as shown in Figure 1. Since very few reported supramolecular cavity designs provide bifunctional activation, cavity catalysis has fared best using approaches such as destabilizing ground states by constrictive binding, guiding molecular collisions to reduce large entropic costs (e.g., pericyclic reactions), and broad, undirected coulombic stabilization of charged transition states [36], for example of cations by hydrophobic hosts [37]. *Directed polarization*, the basis for organocatalysis, is rare in cavity catalysis.

Now, I believe the field of supramolecular catalysis to be on the cusp of putting these two elements – “organization and polarization” or “confinement and dual activation” – together with greater precision. In this perspective, I identify three clear areas for increased focus to achieve this ambition: (1) the development of modular cavities featuring sub-Ångstrom-confined bifunctionality (including frustrated charges); (2) improved access to stable cavities with reduced symmetry via self-assembly by exploiting emergent geometric rules and post-assembly modifications; (3) improved screening and collection of detailed structure–activity-relationship (SAR) data for modular systems to allow systematic design, rational and computational development, and identification of novel activity. These developments require rethinking cavity design, but will be achieved predominantly by synthetic advances, for instance by the internal functionalization of cavities with bifunctionality – chemical groups that simultaneously activate nucleophiles and electrophiles or otherwise stabilize charged pairs. Herein, I argue that an underexplored cavity type, *robust organic cages* [38–47], are uniquely positioned to facilitate these advances.

## Outline and Overview

The aim of this perspective is twofold: (1) to briefly review the state of the art of cavity catalysis, highlighting the catalytic concepts of *organization* and *polarization*, and anticipating future developments; and, (2) to introduce *robust organic cages* as functional enzyme mimics, and highlight how their unique features might advance cavity catalysis and provide more realistic enzyme models for studying electric field catalysis and enzyme dynamics. The wider history of supramolecular and cavity catalysis [3,13,15–19,21,48–49], and catalysis using confined transition-metal catalysts [50–52], dendrimers [53] or synzymes [54], micelles [55] or vesicles [56], catalytic antibodies [57–59] or molecularly imprinted polymers (MIPs) [60] has been discussed elsewhere and will not be covered.

## Perspective

### Cavity catalysis: current state of the art

#### Functionalized macrocycles

**Figure 2:**
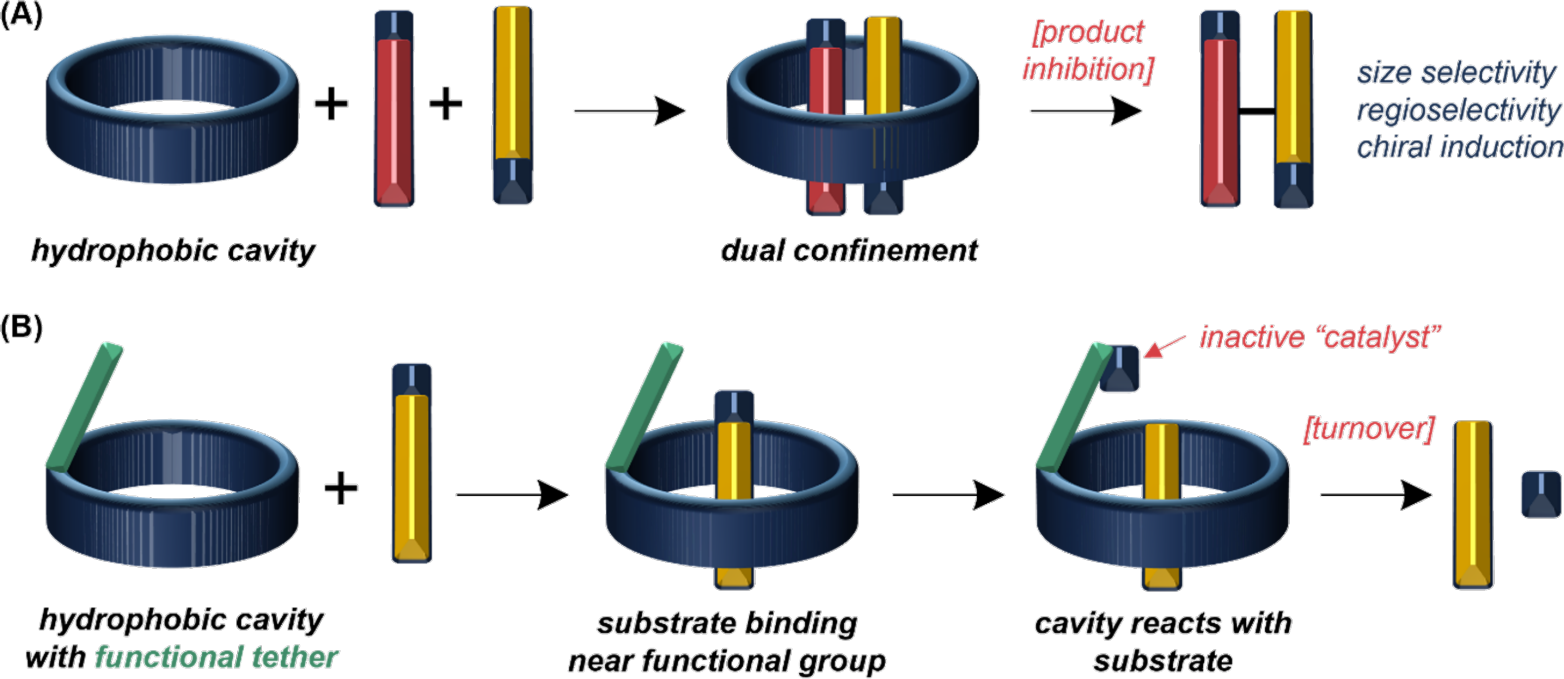
Typical catalysis modes using macrocycle cavities performing (non-specific) hydrophobic substrate binding. (A) Macrocycles that confine two reagents close together to alter reactivity. (B) Macrocycles with functional sites that react with bound substrates directly.

Since Cramer’s work with cyclodextrins [61–63], there has been significant interest in using macrocyclic confinement to modulate substrate reactivity [64]. Cyclodextrins, cucurbiturils, cavitands, and calixarenes are representative [64–70], and typical features of these macrocycles are high symmetry and a hydrophobic cavity with polar edge groups. They tend to be synthesized as covalent cyclic oligomers in mixtures of ring sizes, either by enzymatic synthesis (cyclodextrins) or thermodynamic synthesis, and are used after separation of the different ring sizes. Since the macrocycles are generically hydrophobic on the interior, they can perform catalysis by dual-confinement of two hydrophobic substrates from water (Figure 2A) [71–73], or by binding a hydrophobic substrate and holding it close to a functional(ized) rim (e.g., as in cyclodextrins) that performs a reaction (Figure 2B) [74–79]. These effects are driven mostly by effective concentration/molarity (i.e., proximity of reacting groups) with little [73], if any, transition-state binding [36,80–81]. Thus, these macrocycles depend on the catalytic concept of *organization*; *polarization* is a minor contributor. Size-exclusion and regioselective outcomes are possible [56,82–85], and symmetric arrays of chiral units (like cyclodextrin) can promote enantioselectivity [76], although turnover from augmented macrocycles is not always achieved [26]. The affinity of generic hydrophobic pockets for a hydrophobic product often leads to product inhibition, since binding a single large product is entropically favored over binding two smaller reagents.

**Figure 3:**
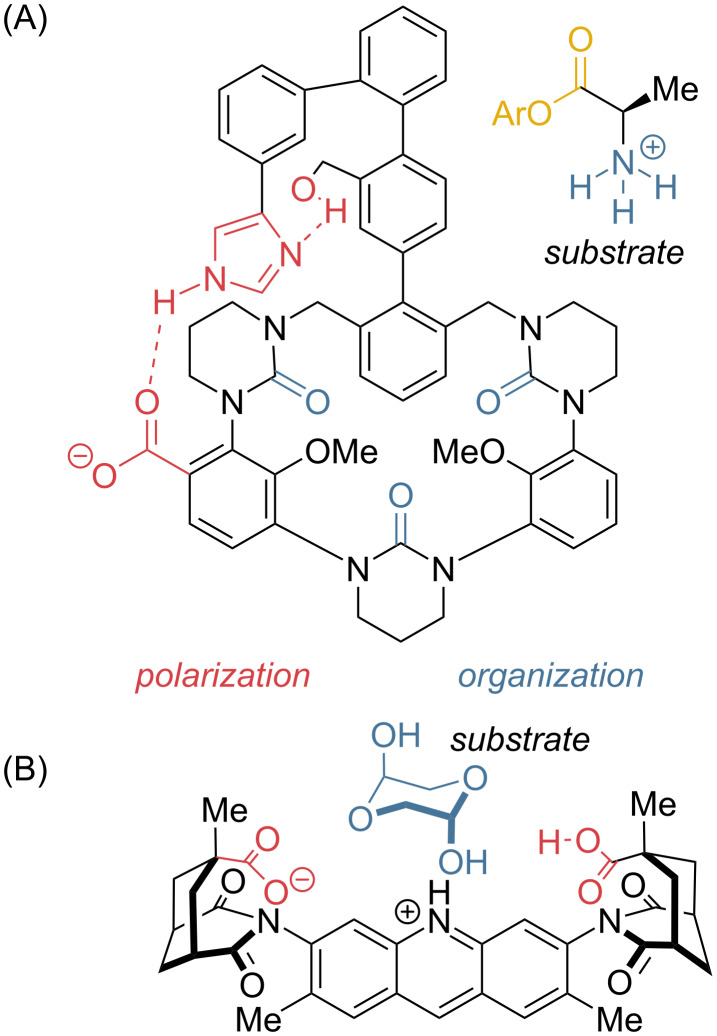
(A) Cram’s serine protease model system [87–88]. The macrocycle showed strong substrate binding (organization), but the model did not show strong acceleration for a hydrolysis reaction. The “organized” catalytic triad (red) was hoped to activate the benzyl alcohol nucleophile, but it does not activate (*polarize*) the electrophile (substrate ester group). (B) One of Rebek’s “clefts” [108–109]. The rigidly organized carboxylic acids both bind the substrate and the transition state for hydrolysis of an acetal.

Also in the category of (functionalized) macrocycles are large enzyme models, such as those reported by Cram [65,86–88], Breslow [74–75,89–92], Diederich [93], and others [94–96], constructed by (often laborious) linear synthesis to afford more elaborate combinations of macrocyclic cavities adorned with functional groups (Figure 3A) [97–98]. These grand “set-piece” enzyme models typically showed only modest catalytic enhancements for enzyme-relevant reactions like the hydrolysis of activated esters, and so mostly contributed to the view that enzymes do not work simply by bringing substrates arbitrarily close to a potentially reactive group [99–100]. One rare but important exception is Breslow’s use of two tethered cyclodextrins to locate hydrophobic ester substrates next to a metal ion. Breslow’s catalyst accelerates the hydrolysis of esters and phosphodiesters by 10^5^–10^7^ by electrophilic activation of ester and nucleophilic activation of water or peroxide at the metal ion [101–102]. The takeaway message is that *polarization* is most effective when it is *bifunctional*. In enzymes, there is never just a nucleophile – there is always a metal, “oxyanion hole”, or “proton wire” [103–106] or equivalent to balance the electron redistribution, which may explain the low activity of Cram’s model serine protease in Figure 3A [107].

*Polarization* was addressed by the creative “molecular clefts” of Rebek [108–109]. Although not a cavity per se, clefts are linear molecules able to direct functionality toward each other in a relatively rigid pincer orientation. One example featuring a pair of antipodal carboxylic acids demonstrated acetal hydrolysis catalysis (Figure 3B) [110]. However, as linear molecules, the clefts are difficult to develop past fixed 2D orientations. Envious of the contemporary progress of catalytic antibodies, Rebek lamented the slow progress in these systems, concluding: *if catalytically useful functionality can be introduced…[to the antibodies] …model systems such as ours may become the dinosaurs of the 1990s* [108]*.* Chemists thus turned toward scaffolds that were easier to access [36,111].

#### Self-assembled containers, capsules, nanoreactors

**Figure 4:**
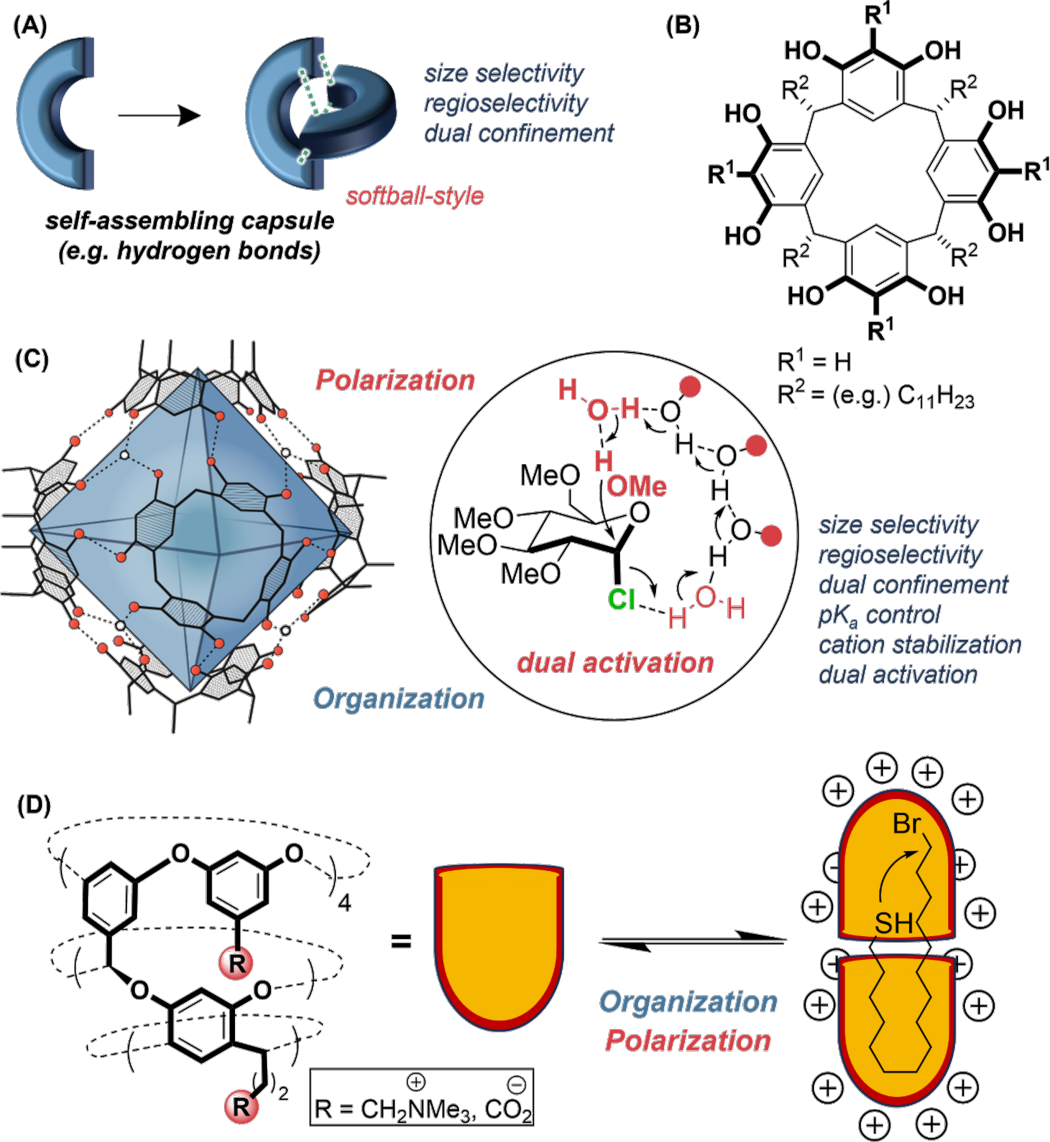
(A) Self-assembling capsules can perform hydrophobic catalysis [116–117]. (B) Resorcin[4]arene building block. (C) Hexameric resorcin[4]arene-based capsules [122] include structural water. The cavity can stabilize cations (substrates or transition states) and perform catalysis using dual activation of a nucleophile and electrophile in a glycosylation reaction [105]. (D) An externally charged cavitand promotes charge-stabilized nucleophilic substitution reactions of hydrophobically encapsulated substrates [136–137].

In the 1990s, Rebek popularized “softball”/”tennis ball” reactors [112–114]. These “capsules” [115–117] are two or more molecules that self-assemble via hydrogen bonds to create an internal cavity (Figure 4A). Since the parts of the capsule are dynamically assembled, substrates and products are able to enter and exit via partial disassembly of the capsule [118], with the only requirement being that they must fit inside [119]. Many of these capsules perform simple hydrophobic catalysis as for the macrocycles discussed in the previous section [115], and so remain prone to product inhibition [113], though not exclusively [120]. Size- and regioselectivity are possible [121]. As with the two identical halves of a tennis ball/softball, it is harder to conceive unsymmetric versions. Recent advances using hexameric resorcin[4]arene-based capsules [122–124] (Figure 4B and C), notably by Tiefenbacher [105,125–133], have demonstrated substrate-controlled selectivities that vary from the bulk phase due to the stabilization of cations in a size-selective space [105,126,129–130,132,134]. A key advantage is that these capsules have been made on multi-hundred-gram scales and can be recycled [132]. Additionally, control of structural or bound water by the capsule [105] and properties such as a lowered p*K*_a_ inside [125] demonstrate a rare example of a cavity-promoting catalysis via both “organization and polarization” (Figure 4C), including dual activation [105], albeit with vague directionality (the substrate can be productively oriented in many conformations). This directionality might be advanced by new capsule types. For instance, the window[1]resorcin[3]arene capsule type [135] demonstrates that the symmetry and properties of traditional symmetric capsules can be modulated, although precise control over the assembly remains difficult. Finally, we must mention the recently reported cavitand capsules of Gibb, who has generated large electrostatic effects by functionalizing the capsule exteriors with charged groups (Figure 4D) [136–137]. Observed rate accelerations for capsule-promoted nucleophilic substitution reactions demonstrate significant enthalpic stabilization of the transition state attributable due to the proximal electrostatic potential, even when the charges are flexibly arranged, remote, and solvated in water.

#### Metal-organic cages

**Figure 5:**
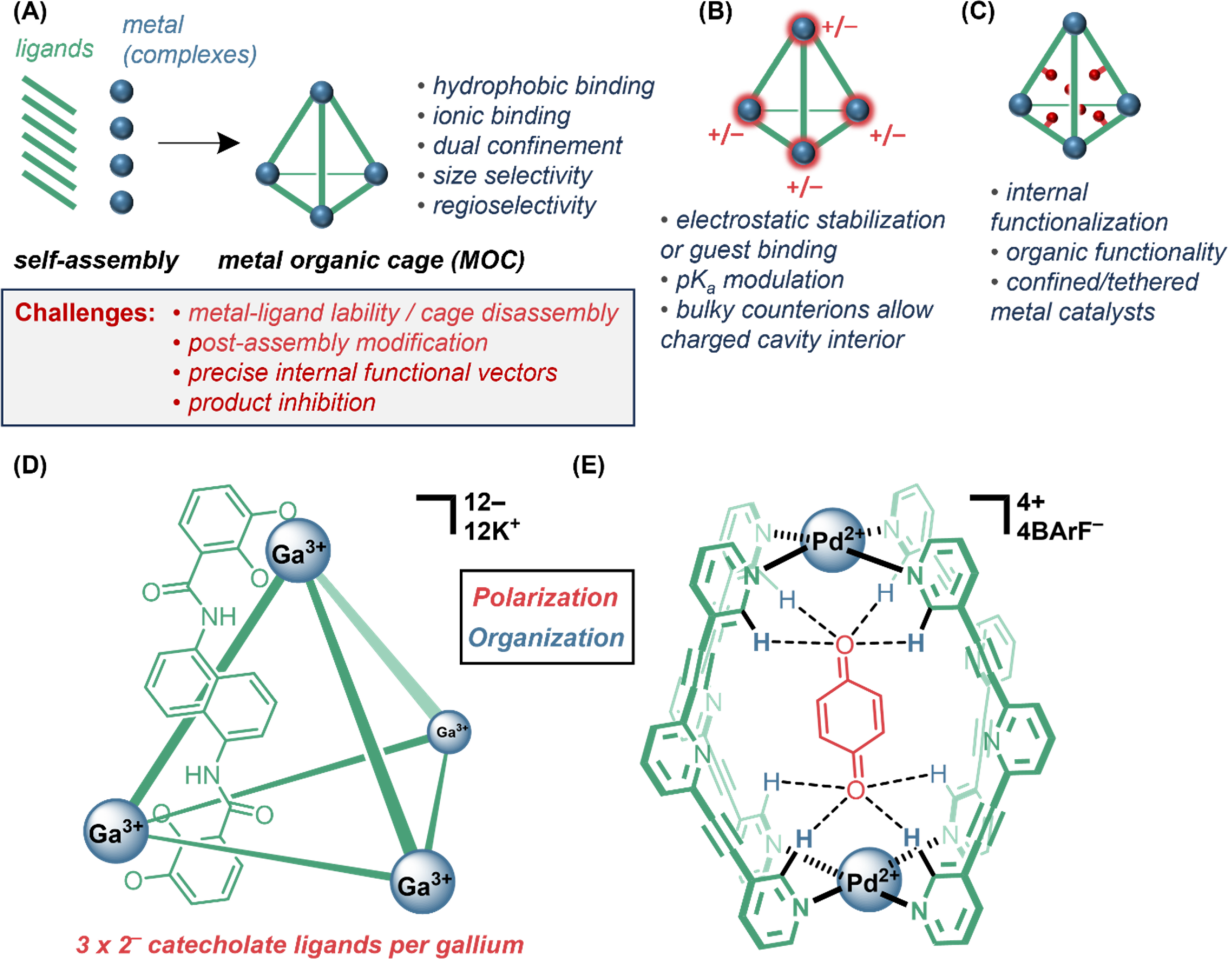
(A) Metal-organic cages and key modes in catalysis. (B) Charged metals or ligands can result in +/− internal charge. (C) Endohedral functionalization of cavity (e.g., with organic groups). (D) Raymond and (E) Lusby-type catalytic MOC systems demonstrate extents of *polarization* and *organization* in catalysis.

The exploration of metal-organic cages (MOCs), also known as supramolecular coordination cages (SCCs), as catalysts is thriving [22,36,138–141]. The reversible bond formation possible in metal–ligand bonding provides chemists with a shortcut to access 3D scaffolds. When well-designed linkers and metals are combined, discrete cages emerge as the thermodynamic product (Figure 5A) [22,142–144]. Typically, rigid linkers are required to enforce geometry, although a “weak-link” approach has been reported [145], and flexible cages are known [146–147]. Application of this “directional bonding” concept [148–150] to synthesize macrocycles and cages was driven by Fujita [151–154] and others [150,155], and the MOCs can then be screened as catalysts, sometimes under the moniker “enzyme mimic” [22,138]. MOCs are typically soluble in polar organic solvents or water [156–157], and so their dynamics can be studied using solution-phase techniques [22]. Isolation from solution is not always possible, since their dynamic nature can make them sensitive to concentration. As for the macrocycles discussed above, dual confinement/encapsulation [36] and the hydrophobic effect dominate the origin of catalytic rate enhancements [158–159]. To avoid product inhibition, model reactions that increase molecularity (A → B + C) or that generate weakly interacting or less hydrophobic [160] products have been popular, including hydrolyses, ring openings, and rearrangements [22]. These reaction classes have been discussed [24–25,140]. Cavity-directed changes in ion-localization [161–162] and p*K*_a_ are effective [37,107,163–164], and size-selectivity [36,165–167] and constriction (ground-state destabilization) are also possible [140,168–170]. The metals can sometimes participate in redox catalysis [171], and may be stabilized by the cage structure [160,172–174]. The organic part of the MOC has also been levied as a hydrogen-bond donor to activate an electrophile [175]. In terms of *polarization*, since cages are invariably charged [36], a few MOCs have demonstrated charge stabilization of transition states (Figure 5B). A key example uses the highly successful Raymond gallium-based cages, exploited by Raymond, Bergman and Toste [21,37,107,155,165,168–169,176–179], which have a “−12” charge in the framework, and can stabilize cationic species (*polarization)* (Figure 5D). However, for reactions in which the charge doesn’t change much between ground state and transition state, catalysis is again predominantly entropically driven inside the cavity, for instance by constriction (*organization*) [36,180–181]. Likewise, the cationic cages of Fujita [151,154,160,170,172,182–189] can stabilize anionic species [184]. The work of Lusby [166,190–196] is notable for using the metals in the cage framework to polarize adjacent aryl–hydrogen bonds in the ligands by enough to coordinate and activate substrates (Figure 5E) [190]. Here, the use of bulky counteranions reinforces the cationic cage interior, which is enhanced further by use of lower polarity solvents, and the p*K*_a_ of confined substrates can vary by an estimated 6 orders of magnitude [195]. The successful Lusby and Raymond-type catalytic manifolds are rare instances of use of both “organization and polarization” approaches to catalysis. A valuable analysis of catalytic modes has been performed [140].

Because MOCs are simple to assemble, modular functionalization of the periphery, sometimes internally projected [164,197–198], has been explored to alter the nature of the cavity (Figure 5C). However, the resulting groups tend to be flexible or loosely oriented and therefore contribute mainly effective molarity effects to catalysis, rather than the more rigidly preorganized functionality required for transition-state binding. If it is easier for a cage to flex [146] or disassemble than to withstand the strain of a transition barrier, the entropic probability of effective transition-state binding is reduced (one can imagine trying to squash a rock with soft tweezers – the tweezers will preferentially bend before breaking the rock).

The trade-off of rapid synthesis of MOCs by self-assembly is that they can lack robustness, and so catalysis conditions have to be mild enough to avoid cage disassembly [22], although compartmentalization of contrasting reactivities is possible to avoid such incompatibilities [189,199–201]. Likewise, the lability of metal–ligand dative bonds can make post-assembly modifications of MOCs challenging [202–203] – for instance it is difficult to lock the dative bonds in place, and reactions that transform the ligands can result in changes in cage topology distribution [204]. Self-assembly can also prescribe limitations on the symmetry of cages [36]. Notably, Clever has recently reported the first Pd_2_(ABCD) MOC [205], which may signal the possibility of using low-symmetry cavities [206–215] for catalysis. To be successful here, tough challenges must be overcome. First, researchers will need to understand better how emergent geometric rules stabilize low-symmetry cavities. This ties in with a second challenge: ensuring that marginally stable low-symmetry cages do not rearrange around the chosen substrates during catalysis. In contrast, strained cages without alternative minima that can relax to bind a transition state may be a productive avenue of research. Related to this conundrum is the recent interest in dynamic MOCs, which show promise in systems chemistry [200]. “Switchable” metal-organic cages [212] use a stimulus like light to change ligand geometries. This often triggers disassembly since new geometries can lead to new thermodynamic minima, though where geometric changes are tolerated within the original structure the stimuli can trigger guest release [216] and even switch catalysis on and off [196]. MOCs containing peptides in the edge pieces also look promising to direct catalysis [217].

#### Extended frameworks

**Figure 6:**
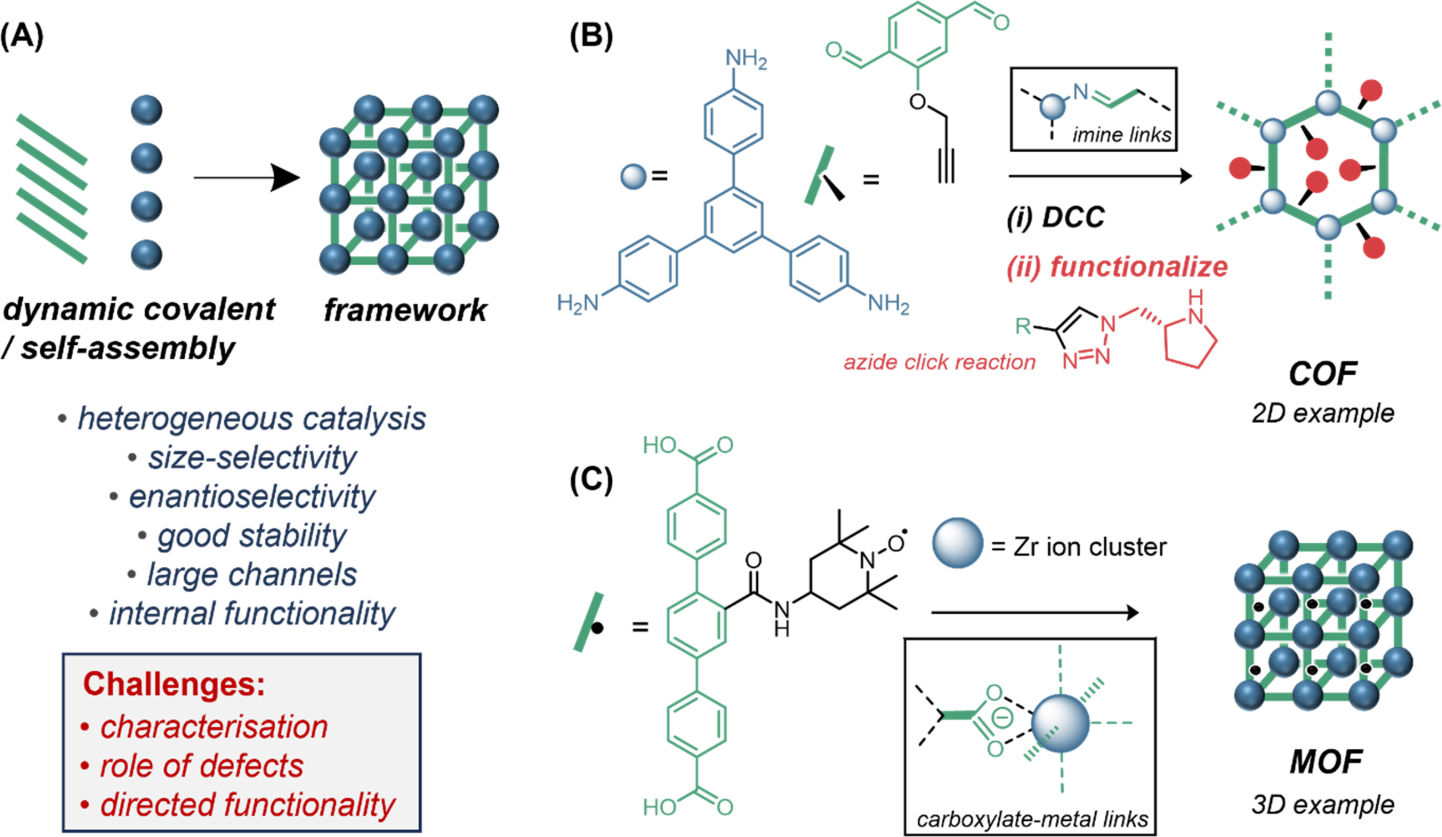
(A) Frameworks (MOFs, COFs) can be catalysts. (B) Example of a 2D-COF, assembled by dynamic covalent chemical (DCC) self-assembly followed by click-chemistry functionalization, containing chiral residues for organocatalysis. (C) Example of a 3D-MOF, comprised of metal clusters linked by dicarboxylate linkers, containing TEMPO ((2,2,6,6-tetramethylpiperidin-1-yl)oxyl) residues for catalysis.

Metal-organic frameworks (MOFs) [218–219] and covalent organic frameworks (COFs) [220–224] are well-studied as heterogeneous catalysts, and since they are the lattice versions of MOCs and organic cages (Figure 6A) we mention them briefly for completeness. The catalytic properties of MOFs compared to MOCs have been discussed [22]. As heterogeneous multisite catalysts, MOFs and COFs are harder to compare to the other “enzyme mimics”, and many just operate as solid-supported versions of existing catalytic motifs [225–226]. For instance, the frameworks must retain channels to allow substrate/product ingress and egress and so the resulting cavities are often quite large and channels can deform if solvent is absent [227]. Lattices can also contain defects, which may affect catalytic activity unpredictably [228]. Nonetheless, macroscale structures, like those that arise from the stacking in 2D COFs, can contribute to catalysis, for instance dense arrays of aligned C–H bonds can provide CH–π interactions in Diels–Alder catalysis [229]. Methods to study the structural detail of catalysis in frameworks remain limited, and crucial techniques like solution-phase NMR are rarely useful [22]. Enzyme encapsulation [230], and electro- and photocatalysis are known [228], and chiral and low symmetry MOFs are an exciting avenue, although the synthesis and characterization (particularly crystallization) of low-symmetry structures remains challenging [227,231–232]. Likewise, COFs hosting chiral organocatalysts are known (Figure 6B) [226,233]. Frameworks are well-suited to hosting opposing reactive functionalities (e.g., acids and bases) and multiple reactive sites can be introduced into a single rigid framework [234], allowing the telescoping of several catalytic steps. However, in terms of *organization* and *polarization*, the challenge is to position within porous materials catalytic motifs (Figure 6C) with sufficient preorganization to catalyze new reactions, better stabilize transition states, or provide new selectivities. The study of discrete, soluble cavities in solution before translation to lattice analogues might prove a fruitful avenue.

#### Covalent organic cages

**Figure 7:**
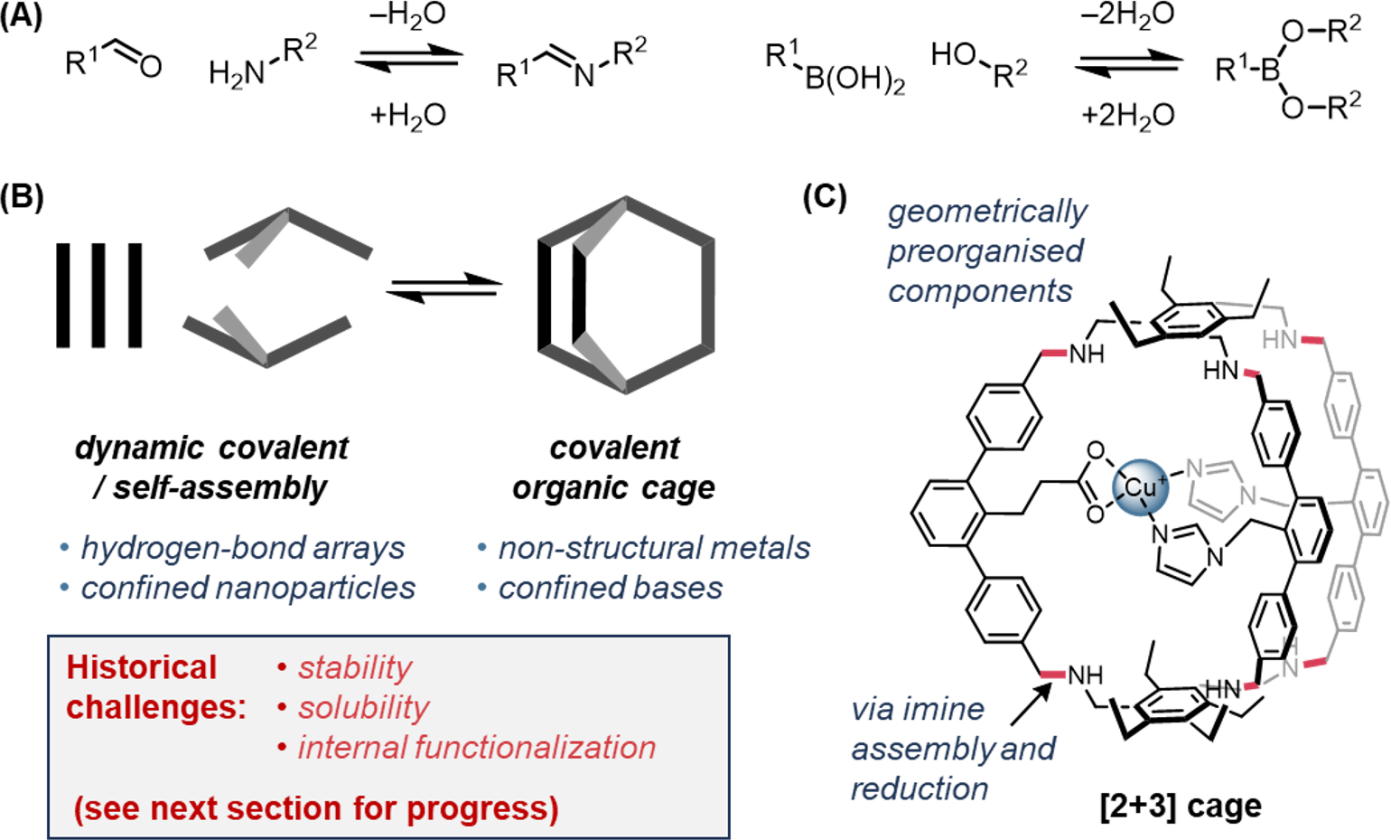
(A) Examples of dynamic covalent chemistry used to synthesize organic cages. (B) Organic cages are gaining traction as soluble discrete organic cavities with catalytic potential. (C) Control of the coordination sphere of a metal in a robust organic cage pMMO (particulate methane monooxygenase) mimic by Otte [44].

Covalent organic cages [235] are any discrete assembly, at least bicyclic in connectivity, whose minimal structure is comprised of covalent bonds. Organic cages have historically been synthesized using irreversible bond formation [236–248], sometimes with a template [45,249–250]. Following the popularization of dynamic covalent chemistry in the 1990s [251–255], macrocycle and cage synthesis using reversible reactions [256–259] like imine formation (Figure 7A) have led to advances in the synthesis of COFs [223–224] and discrete organic cages [260–284], notably porous organic cages (POCs) since 2008 [285–289], by dynamic covalent “self-assembly” (Figure 7B) [258]. In these cases, although covalent bonds are formed, bond formation is still self-directing and self-correcting, and the term “self-assembly” [290–292] often used [286,293–296]. Products can be thermodynamic or kinetic, but are often isolated by precipitation due to low solubility [297], though many have useful solubility [288,298]. Since POCs typically have little internal functionality in their cavities, they have found far greater utility as porous solids (e.g., for gas uptake or separations) than as catalysts [287–288,298–314]. Catalysis with organic cages has only emerged in the past few years [20,70,235,315], and true organocatalysis is exceedingly rare [316]. Instead, catalytic systems tend to be composed of cavities that increase substrate solubility [317], or host nanoparticles [318–326], metals [44,327–328], photoactive groups [329–331], superbases [332–336], or non-specific arrays of hydrogen-bond donors [337]/acceptors [338]. Advances in post-assembly modifications [339] have recently allowed stable organic cages featuring endohedral (internally directed) functionality [340–341], or metals [342–345], which show early promise for low-symmetry cavities with catalytic potential [42–44,340]. Notably, Otte has used semi-stepwise self-assembly via imine formation/reduction to access a robust organic cage with reduced-symmetry and internal functionality able to chelate a copper(I) ion, which can act as an oxidation catalyst (Figure 7C) [44]. The long-standing challenges of synthesis, stability, solubility and internal functionalization are beginning to be tackled, and the remainder of this Perspective will discuss specifics of these hard-earned advances and the opportunities they unlock for cavity catalysis.

### Perspective: on new directions for organic cages

#### Functionalizable, stable, soluble organic cages as cavity catalysts

Surprisingly, despite the fact enzymes are predominantly organic molecules performing organocatalysis [100], cavity-based organocatalysis remains under-represented in the supramolecular literature [4,70]. My laboratory has therefore attempted to understand the reason for this deficit, and to identify solutions [38–40]. Firstly, cavities containing oriented functional organic groups (like those that participate in enzyme active sites) remain conspicuously rare [340]. One reason is the requirement for structural directional bonding in self-assembly strategies, which limits available bonding vectors for precise internally directed functionality [340]. Indeed, a recent perspective [21] identified two approaches to site-selectivity in supramolecular host catalysis – (i) using the host as a “protecting group” to direct reactivity external to the host [187,346], and (ii) confinement of a transition-metal catalyst to take advantage of the restricted environment of the host [51–52] – neither of which resembles the enzyme-like possibility of a true active site (binding a substrate in an orientation that directs internally catalyzed reactivity) [347–348].

The examples of cavities with functionality discussed above, from Cram’s “full serine protease model” [87], to MOCs with flexible peripheral groups [349], to frameworks with internal proline organocatalysts [222,234] all suffer from the same limitation: they all fail to rigidly organize sufficient bifunctional groups to obtain clear transition-state binding – a hallmark of enzymes and organocatalysts [107,180].

**Strategy towards organocatalytic organic cages:** My laboratory has levied the following design criteria in the pursuit of organocatalytic organic cages: (i) efficient synthesis, ideally by self-assembly; (ii) soluble and stable in organic solvent and in the presence of reactive reagents; (iii) extreme preorganization of functionality in a cavity; (iv) the lowest possible symmetry.

We quickly identified the triptycenyl-based imine cages of Mastalerz [301] as a strong starting point because: (i) they offered efficient, modular assembly; (ii) all of the complexity could be confined to the privileged triptycene motifs, which would present rigid internal vectors into the cavity for selective substrate and transition-state binding, and for which extensive synthetic development is known [350–355]; (iii) they are the lowest possible symmetry polymacrocyclic structure (removing one edge piece will result in a macrocycle) [356–357], meaning ordered asymmetric structures would require fewest augmentations. Otte has levied similar techniques to generate soluble amine-based organic cages with the same symmetry possibilities, using the three edge pieces to provide internal vectors to generate “catalytic triad” mimics [42–44].

**Figure 8:**
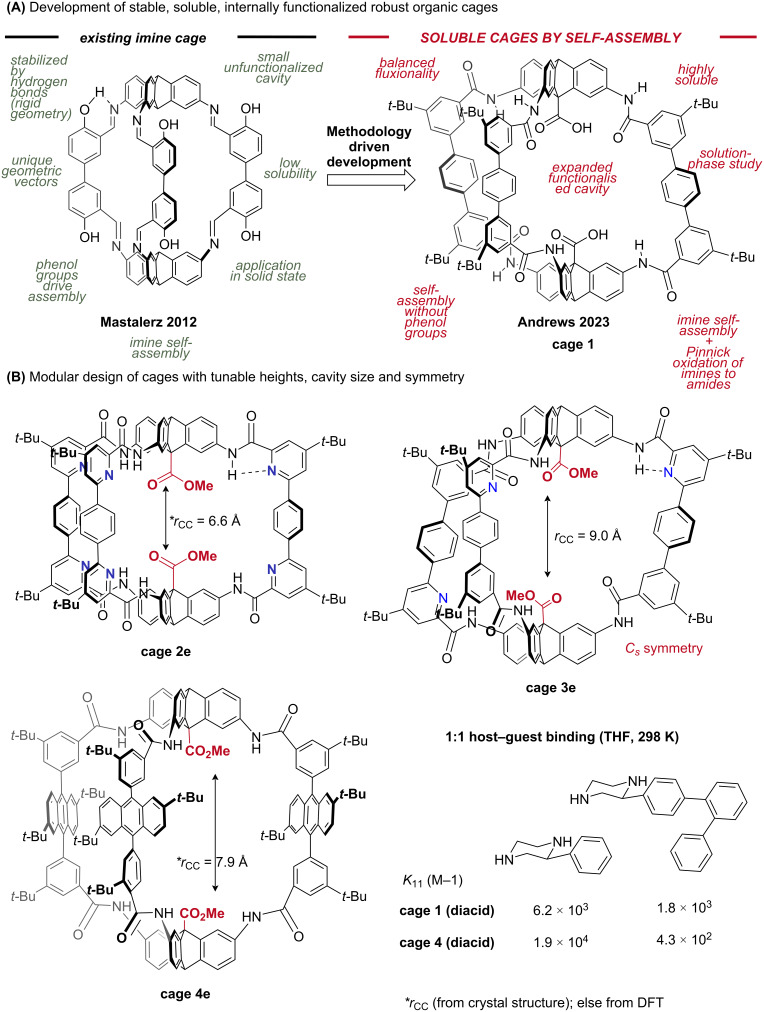
(A) Design and development of soluble, functionalized, robust organic cages. (B) Examples of modular structural changes and properties. Ester hydrolysis of cages **2e**–**4e** gives the diacid analogues **2**–**4**.

**Synthesis of robust organic cages:** When we entered the field, it quickly became apparent that one reason functional organocatalytic cages had not been reported is the synthetic challenge: our chosen cage frameworks [300,302], at least, were poorly soluble [297], and required development to exploit them in the solution state (Figure 8A) [38]. To capture both stability and solubility, we turned to Mastalerz’s post-functionalization chemistry [286,300,304,306,358–362], in which imines are oxidized by a Pinnick oxidation to amides [286,360,363–364], an approach gaining popularity [46,262]. Importantly, we were able to adapt this chemistry to work in situ, allowing soluble metastable imine frameworks to be trapped as amides [38]. The adapted cages were soluble, and were now stable enough to be purified by gel-permeation (size-exclusion) chromatography (useful when precipitation is not possible), which is typically not possible for imine [314] or metal-coordination cages [156,202,365]. Further, the robust amide cages could withstand harsh conditions such as ester hydrolysis, allowing access to the key acid-functionalized cages [38] that mimic aspartyl proteases and glycoside hydrolases. Otte has employed reductive amination to stabilize imine cages, and the resulting amine cages gain solubility from increased flexibility at the cost of losing some structural rigidity [42–44].

**Figure 9:**
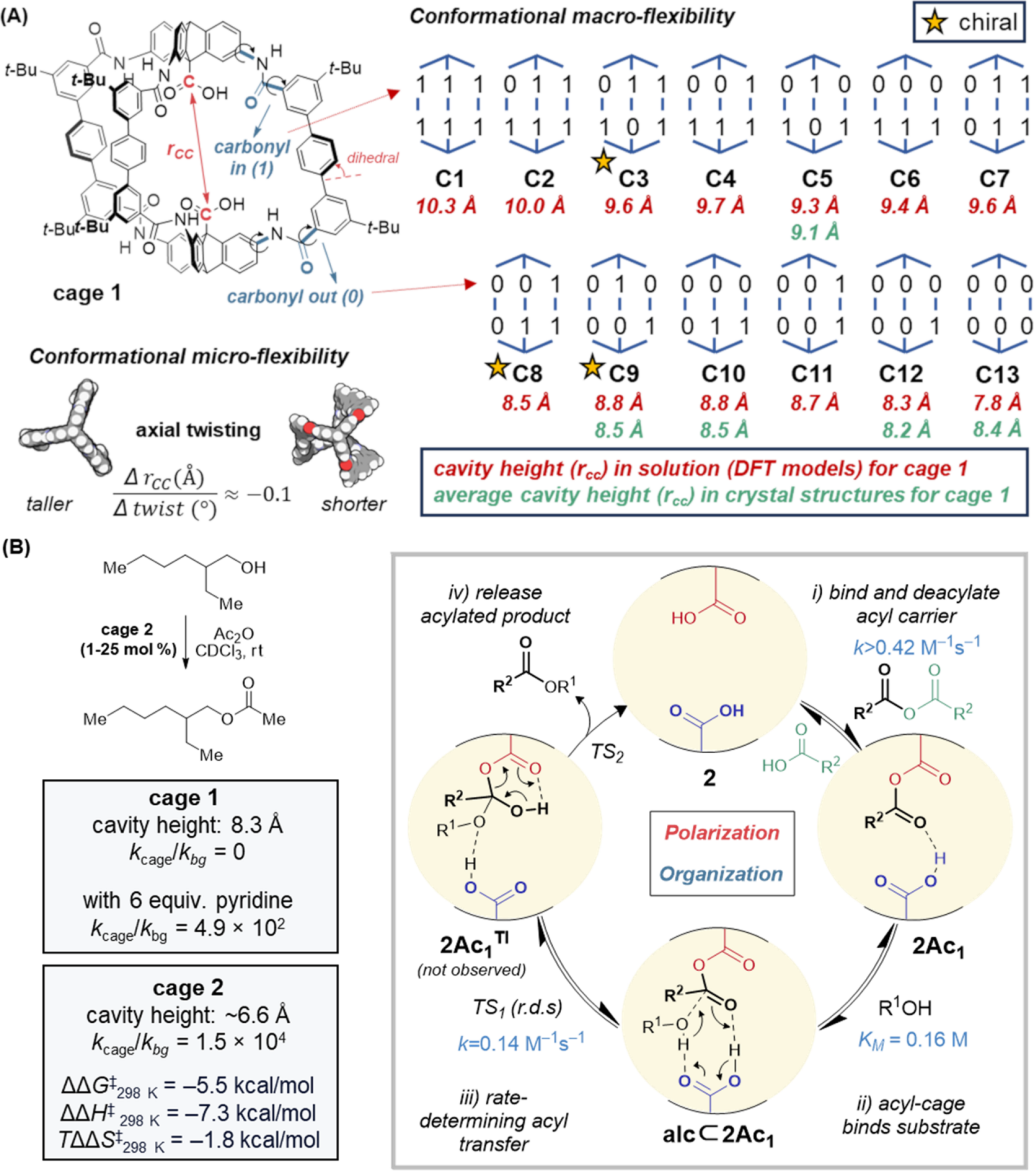
(A) There are 13 metastable conformers (symmetry-corrected) for cage **1** due to permutations of amide conformations. Cages **1**–**4** have cavity heights *r_CC_* that change with carbonyl orientation (“macroflexibility”). Additionally, cavity heights can change within a conformation due to cage axial twisting (“microflexibility”). For cage **1**, crystal structures have been measured for the more stable conformers **C5**, **C9**, **C10**, **C12**, and **C13**. (B) Organocatalysis performed by cage **2** (which exists exclusively in the **C13** conformer) is a clear example of *polarization* and *organization*. The data for cage **1** suggest cavity height (balanced flexibility) is important for the catalytic rate.

**Metastable conformations – programming cavity shape and symmetry:** Unlike non-covalent/dative assemblies, covalently linked cages can incorporate greater bond strain, and therefore the linkers themselves can contribute to cavity shape and symmetry outcomes. For instance, in our cages [38–41], due to the preference for *N*-phenylbenzamides to be planar, each amide group can be arranged in two metastable orientations: amide carbonyl oriented outward (designated by “0” or “O”) and amide carbonyl oriented inward (designated by “1” or “I”). There are 64 (2^6^) permutations of carbonyl orientations in our cages, of which 13 are unique for cage **1** after grouping by symmetry (and ignoring enantiomers), which we have labelled as **C1**–**C13** (Figure 9A) [39–40]. For cage **1**, DFT calculations suggests a population consisting of perhaps two major conformers (**C5**, **C9**), with 3–4 minor contributors (298 K, THF) [39–40], but we can control the preference of these conformers by introducing functionality which favors particular amide orientations [39]. For instance, hexapyridine cage **2e** (Figure 8B) shows exclusive occupation of **C13** (all carbonyls out) due to six favorable NH_amide_ *→* N*_pyr_* interactions [41], while trispyridyl containing cage **3e** has three favorable NH*_amide_* *→* N*_pyr_* interactions and so shows a solution-state preference for a low-symmetry (*C_S_*) conformation consistent with **C5** (Figure 8A) [39]. This low-symmetry cage **3e** was designed by taking advantage of the geometric rules discovered studying cage **1**. We have introduced the term *conformational autodesymmetrization* to describe this rational approach to accessing low-symmetry cavities [39]. In this approach, symmetric topologies undergo a natural symmetry-lowering process when the restricted angles possible in a polymacrocycle environment lead to non-symmetric conformations in the pursuit of equal strain distribution. In the case of cage **3e**, the mixed pyridyl/aryl system results in a strong reinforcement of the geometrically preferred **C5** conformer by coinciding pyridyl groups with “out” carbonyls. The result is a lowering of symmetry from *D*_3_*_h_* to *C_S_*. While observations of symmetry-lowering are commonplace in controlled environments [47,264,293,335,366–369], we believe there to be wide-ranging potential across low-symmetry cavity research [205,208,309,366,370–374] if new and more explicit emergent geometric rules can be codified and exploited [39]. We were also able to statistically access a cage with a single internal carboxylic acid group and purify the cage by size-exclusion (GPC) due to the cage robustness [41]. Use of bulky internal groups can statistically bias cage self-assembly of mixed groups, a strategy previously reported [375].

**Versatile characterization in the solid and solution states:** The stable and soluble nature of covalent organic cages allows structural analysis in a way not possible for assemblies [38–39,41–44]. Due to their enhanced stability over imine assemblies, our amide-linked cages are amenable to complex processing (the cages remain unchanged in connectivity across changing solvents and temperature) and therefore can be subjected to automated and high-throughput crystallization studies and, with Szczypiński, Slater, Cooper and co-workers, we were able to isolate all five calculated lowest-energy conformers of cage **1** in the solid state (see crystal cavity heights marked under conformers in Figure 9A) [40]. Meanwhile, cages **1** and **4** have been studied in the solution state as hosts for diamines (Figure 8B) [38–39], guests which would ostensibly degrade host imine cages or metal-organic cages [345]. Indeed, imine cages are rarely viable as solution-phase hosts [263,342–343,345]. We were also able to study the low-symmetry conformation of cage **3** in solution [39]. Due to their stability, we can typically reclaim our cages up to quantitatively after binding or catalysis experiments, often just via a work-up and precipitation.

This breadth of available data opens considerable opportunities in computational and rational design, which depend on systematic access to incrementally varied experimental datasets, which are not always available for metastable systems. Further, without many-electron metals to model [208,376], high-throughput calculations of organic cages are facile [366,377–385], and do not require metal parameterization [140,386–387].

**Catalysis:** Stable, soluble organic cages finally open the possibility of organocatalysis in confined spaces: *organization and polarization*. Hexapyridyl cage **2** was shown to accelerate acyl transfer esterification reactions by more than 10^4^ compared to the background rate (298 K, CDCl_3_) (Figure 9B) [41]. Here, the strongly preorganized pair of carboxylic acids split duties: one covalently activates an acyl group while the other provides bifunctional acid/base activation. Together, they bind the transition state, enthalpically stabilizing the rate-limiting attack of alcohol by 7.3 kcal/mol. The highly ordered transition state in this example, thought to prevent charge buildup through a concerted/synchronous proton-transfer mechanism between bifunctional acid units, highlights a key design criteria differing from the early work on enzyme models: *the transition state is bound strongly, not the substrate.* As a result, in this system the ester product does not inhibit the reaction (instead, accumulating acid inhibits the reaction, likely by interfering with the protic transition state, although this can be mitigated by addition of base). Furthermore, since substrate binding is weak in this system (≈1 kcal/mol), the potential to alter the cage periphery to direct site-selective reactivity inside the cavity is enormous. Because the reaction is oriented precisely with respect to the cage axis, catalysis becomes amenable to rational design, and functional and electrostatic tuning of the cavity are expected to be fruitful and comprehendible. The stability and solubility of robust organic cages in organic solvent highlights a clear niche for mimics alongside enzymes. As well as tolerating higher temperatures and more reactive reagents (“cofactors”), they could perform non-biotic reactions, and even have potential for the design of novel active sites that could be transposed into new designer enzymes for optimization [388–391], since directed evolution requires initial activity to work from.

**Flexibility vs rigidity:** Cage designs often face unstructured criticism for being either too flexible [146] or too rigid [25] to mimic enzymes. This is not the place for a detailed discussion on the nuances of these vague terms in enzymology [392–394], but we hope the following is persuasive. In considering the relative positions of the two active carboxylic acid groups in cages such as **1**, there are several levels of tuning possible in terms of the rigidity. Hexapyridine cage **2** is calculated (and observed) to exist exclusively (>99.9%) in the **C13** conformer (Figure 8B and Figure 9A) [41]. Since the amide groups rarely rotate in this form [41], the structure can be termed rigid. Nonetheless, slight variations in the cavity height can be controlled by engineering the twist along the triptycene axis. For instance, comparing crystal structures of cage **2** and its monoacetylated analogue, **2Ac_1_** (Figure 9B), a decrease of 0.3 Å (5%) in the acid–acid distance (*r_CC_*) is observed along with an increase in twist by 2° (i.e. ≈ −0.1 Å/°) [39,41]. This twist can in turn be controlled by the dihedral angle of the central component of the terphenyl group (Figure 9A) – for anthracene cage **4e**, an increased dihedral angle due to a steric clash is thought to contribute to the increased cage axial twist and reduced cavity height observed in the crystal structure [39]. Thus the cages have “micro-flexibility” of the sort that can finetune enzyme-like transition-state binding, and perhaps even satisfy Sanders, who has lamented the lack of control of rigidity in supramolecular catalysis [25]. In contrast to cage **2**, cage **1** has access to at least 5 conformers by amide rotation, covering a flexibility of perhaps 1.5–2 Å (≈20% increase from the smallest size) [39–40]. This “macro-flexibility” is more akin to large enzyme-like movements that might permit tuning of several consecutive catalytic steps [395], induced-fit binding, product release, allostery [396–397], or signaling. For the acyl transfer reaction, cage **1** is an inferior catalyst compared to cage **2,** which we have postulated is due to the larger equilibrium cage height (increased flexibility) (Figure 9B) [41]. The ability to tune catalytic activity by tuning the cage rigidity, conformation, or dynamics is certainly an advantage rather than a liability [395,398–402]. It is perhaps unsurprising that the balance of rigidity and flexibility [403–404] contributed by the amide links in proteins seems to be well-reproduced in our amide-linked cages.

In summary, the ability to reliably and predictably access stable, soluble, low-symmetry cages, with tuneable functional group projections and tailorable flexibility, and all by dynamic covalent self-assembly, means there are opportunities in robust organic cage research not present in any other type of cavity currently explored.

#### Automation and calculation in cavity synthesis and study

The computational discovery of new materials has advanced in recent years with increased computational power [405]. Imine-based porous organic cages have been a popular choice for study [377,379,406–408], as have MOCs [208,376,386]. Much focus remains on the prediction (and automation) [409] of the formation of cavities by probing combinations of, e.g., amines/aldehydes or metals/ligands to identify structures with clear thermodynamic minima [410]. Although this approach might be forward-thinking in terms of materials access, cost, and scale, without precise property prediction it requires serendipity in terms of function-discovery within a “near infinite design space” [376]. Further, by definition, screening for particularly stable assemblies [405,411] screens out cages with the taut, dynamic properties found in biological systems [100,146,395,401,412]. Therefore, despite the apparent similarity between porous organic cages (solid state) and robust organic cages (solution state), current leading methods in materials discovery are unlikely to discover solution-phase sensors or catalysts without a rational starting point to build from.

Instead, new receptors [413] and catalysts [414–416] will likely arise from improvements in computational rationalization of experimental data [415,417], which in many cases is the only way to understand pure substituent effects (e.g., on catalysis). To enable the validation of this process, experimentalists must collect data relevant to benchmarking computations: binding constants, kinetic barriers, crystal structures. In turn, this process will be enabled by improving automated experimental screening of existing cavities for new activity (sensing, catalysis) [418]. Crucially, improved access to experimental structure–activity relationships of incrementally developed cavities [21] is required to feed rational or machine learning advances. The unique purification possibilities available for robust organic cages mean access to structural families via stepwise synthesis or statistical methods followed by separation is facile, as demonstrated by Otte [42–44] and ourselves [41]. The lack of internal functionality in cavities doesn’t just reduce the possibility of larger *polarization* contributions to catalysis, it also makes property prediction difficult [419] since computational appraisal of nebulous additive effects remains challenging and ungrounded, and difficult to benchmark or validate experimentally. Materials with more precise substrate and transition-state binding modes [41,105], such as those found in robust organic cages, can therefore be readily studied, understood, and improved.

## Conclusions and Final Perspective

In this perspective, I have highlighted the many advantages for studying enzyme mimics unlocked by recent developments in the synthesis of self-assembled, robust organic cages with internal functionality [38–44]. Robust organic cages are under-represented in the supramolecular catalysis and enzyme-mimicry literature, an observation which correlates strongly with the lack of cavity-based organocatalysts. The lack of organic cage organocatalysts is surprising given that the majority of enzymes are organic cavities performing organocatalysis [100]. Notably, we present a call-to-arms for cavity systems with rigidly preorganized transition-state binding motifs – flexible, peripheral motifs around cavities are more likely to organize substrate selectivities than provide large transition-state stabilizations. This follows from the requirement for differential transition-state binding over substrate binding for catalysis [100], and requires cavities that *polarize* in addition to *organizing*, that is, an *organized polarization* [420]. Impactful advances await for researchers that rationally elaborate on the simple, symmetric structures often initially screened for interest. Quantifying patterns found in (automated) screening will be vital to ensure that unintuitive geometric [39,205,207,211,366–367] and thermodynamic rules inform sequential iteration [96,421–422], since the supramolecular landscape is vast, and highly successful systems still tend to be those patiently developed [45,248,423–424] (as Nature has) rather than discovered in a few simple reactions. One is reminded of Rebek’s trite observation that host systems are frequently chosen on the basis of simple synthetic accessibility, briefly screened for activity, and “…the word *design* … much over-used in the publication” [425].

In terms of application focus, site-selectivity in polyfunctionalized (e.g., bio-derived) materials [426–428] is of increasing importance in the search for sustainable feedstocks, and cages that *organize* and *polarize* have advantages in this precise reactivity. The need for *polarization* may be circumvented by incorporating photocatalysts into cages [183,429], which are also likely to provide novel site-selective reactions [21,185,430–433]. Cage structure may also activate photocatalysts [434] or help restrict detrimental photocatalyst deactivation reactions [435].

We also point towards *conformational autodesymmetrization* [39] as a largely ignored strategy to develop low-symmetry cavities using the tools of self-assembly. Mathematical and computational models should accelerate the discovery of stable low-symmetry structures by identifying ligands or linkers featuring mutually reinforcing symmetry-breaking functionality [205]. Robust organic cages are also likely to offer advantages for studying switchable, functionally dynamic and strained systems, since metastable cavities are less likely to tolerate the structural strain that switching can induce [345,436–437].

We end by noting a recent perspective on cavity catalysis, which concluded that: “*How much of a real comparison can be drawn between a simple, highly symmetrical, often hydrophobic pocket of a typical coordination cage host system and the complex, highly unsymmetrical and largely polar environment of an enzyme active site is a moot point… [since]… the mechanisms which an enzyme utilizes to accelerate chemical reactions are in many cases very different to those seen in supramolecular catalysis”* [36]*.* While happy to agree with the description of historical supramolecular catalysis approaches, we also anticipate that structurally tailored robust organic cages will finally allow access to more relevant enzyme mimics, organocatalytic motifs, and functional triads in precise-enough ways to permit a new wave of enzyme model studies, this time to reveal details of electric field catalysis [99,417,438–440] and the elusive roles of enzyme dynamics [395,401,441].

## Supporting Information

File 1 Additional reference annotations.

## Data Availability

Data sharing is not applicable as no new data was generated or analyzed in this study.
