# Supplementary material for: Beyond symmetric self-assembly and effective molarity: unlocking functional enzyme mimics with robust organic cages
Source: Beilstein J Org Chem. 2025 Feb 24;21:421–43. doi: 10.3762/bjoc.21.30 (PMC11878132; doi:10.3762/bjoc.21.30)
Supplement: File 1 — Additional reference annotations. [file Beilstein_J_Org_Chem-21-421-s001.pdf]

BEILSTEIN JOURNAL OF ORGANIC CHEMISTRY

Supporting Information

for

Beyond symmetric self-assembly and effective molarity: unlocking functional enzyme mimics with robust organic cages

Keith G. Andrews

Beilstein J. Org. Chem. **2025,** *21,* 421–443. doi:10.3762/bjoc.21.30

Additional reference annotations

License and Terms: This is a supporting information file under the terms of the Creative Commons Attribution License (https://creativecommons.org/licenses/by/4.0). Please note that the reuse, redistribution and reproduction in particular requires that the author(s) and source are credited and that individual graphics may be subject to special legal provisions.
The license is subject to the *Beilstein Journal of Organic Chemistry* terms and conditions: (https://www.beilstein-journals.org/bjoc/terms)

In order to facilitate the expeditious locating of supporting claims referenced in the main text, we include an annotated reference justification. The aim of the justification is to aid transparency, and save time for readers. The trail includes:

- details of which tables etc. quoted values are taken from;
- quotes from the original manuscripts to allow instant localisation of supporting claims using modern computer search functions;
- author comment on groups of references;
- longer footnotes;
- examples of counter-references disputing a claim made in the manuscript, where appropriate;

**Introduction**	
The unique environment in enzyme active sites can accelerate chemical reactions by as much as 10^{19} compared to the uncatalyzed reaction in water.<br><br>rate-accelerations (10^{19}),[1]	Wolfenden and Snider describe the range of enzyme rate-enhancements in Figure 5 as 10^{7}-fold to 10^{19}-fold.
It is for this reason that I have sought to introduce supramolecular approaches into my organocatalysis.[2]	The preorganized “pendant” phenol in our Mitsunobu catalyst contains perhaps the simplest example of enzyme-like organization.
Organization, the control of the position(s) and orientation(s) of reacting molecules, has been achieved historically in supramolecular chemistry using “pre-organized” catalyst designs (*vide infra*). More recently, *organization* has been mooted by List as a unifying concept across many fields of selective catalysis under the term *confinement* [3], a term borrowed from heterogeneous catalysis.	We have found the List definition of “confinement” to either mean organisation, or to also include polar matching.<br><br>• “confinement,” the shaping of a catalyst's active site.<br>• Enzyme-catalyzed reactions often feature the additional aspect of the chiral confinement, i.e., the selection of one enantiomer over the other.<br><br>Example meaning organisation: “The combination of a confined active site with reactive functional groups has historically led to rapid advancements in catalysis”<br><br>Example meaning organisation: “This notion led to extensive applications and can often provide a good explanation for observed selectivities based on the ground state conformations of the reactants. In the remaining part of this perspective, we would like to show that the key principles of “confinement,” which lead to the emergence of effects such as shape- and size-selectivity, prevention of side reactions, substrate preorganization, and stabilization of reactive intermediates can indeed be recognized for certain small-molecule catalyst systems. We conclude that the ideal confined catalyst shapes a specific reaction volume that is exclusively complementary to the transition state of the desired transformation by specifically limiting the degrees of freedom of the interacting reactants. It has to be noted that this definition of confinement

	is, to some extent, different from the bare description of second coordination sphere effects, which has been used for heterogeneous and supramolecular catalysts."<br><br>Example where confinement is suggested to mean both organisation and polarisation: "In general, basic atomic properties drastically change upon confinement within the catalyst framework, leading to effects such as increased excitation energy and lower polarizability, which can be explained using the "particle in the box" as a simplified model.[3] For larger molecules, spectroscopic evidence for an intrinsic decrease in the π-π∗ gap in aromatic hydrocarbons such as naphthalene and anthracene has been shown and was computationally supported by Márquez, García, and Corma.[4,5,6,7] More recent studies, however, revealed that the pure effect of confinement rather leads to an increase in the HOMO-LUMO gap and point toward the specific properties of electrostatic stabilization within the active catalytic site—a unique consequence of the confined active center.[8,9]"
the unifying concept for *polarization* is that of "cooperative[4] bifunctionality":	
dual activation (e.g. the simultaneous activation of nucleophile and electrophile).[5-12]	Examples of works involving dual activation, including "bimetallic catalysis", "ambifunctional catalysts", "bifunctional/ Multifunctional catalysts".
Supramolecular chemists have made significant advances in cavity catalysis[13-24]	Reviews and textbooks on supramolecular catalysis
albeit slowly[25] – but have predominantly achieved *organization* and *polarization* independently.[26]	Sanders' review lamenting slow progress in supramolecular catalysis.[25]<br><br>Scrimin reviews the very few synthetic species deemed true "enzyme mimics".[26]<br><br>"…the design strategy of enzyme mimics is often minimalist, relying on those features that are deemed essential in enzymes for catalytic efficiency and neglecting all the rest."<br><br>Table 1 shows that the source of catalysis in these cases are often limited to a subset of an enzyme's approach.
This even appears to be a strategy: *"the search for supramolecular reactors that contain no catalytically active sites but can promote chemical transformations has received significant attention, but it remains a synthetic challenge."*[27]	Quote, found in the abstract
The vague term "confinement" is sometimes used as a catch-all explanation for the property changes that arise within a cavity environment, often in the context of zeolites.[28-32]	"An *electronic confinement concept* has been proposed based on the concept that the molecular orbitals (MO) of the adsorbates inside the zeolite pores are not extended over all the space, as they are in the gas phase, but instead are forced to limit within the zeolite walls. This "*boxing effect*" is stronger as the size of the

	confined guest approaches the zeolite cage dimension, producing an energy increase of all MOs particularly those which are more diffuse”[29]<br><br>“It is well-known that spatial confinement has an influence on the electronic structure, energy spectra, and chemical reactivity of atoms and molecules.”[30]<br><br>*“Ab initio* calculations showing frontier molecular orbital energy modifications of a guest molecule when located inside a microporous zeolitic cavity are presented.”[32]
In contrast, modern descriptions of enzyme active sites speak of *organized polarization* rather than “confinement,” specifically positing that oriented electric fields rule transition-state stabilization for many reactions.[33-35]	Warshel has championed this theory for decades.
and broad, undirected coulombic stabilization of charged transition states,[36]	Lusby and Spicer discuss coordination cages and the role of coulombic stabilization
for example of cations by hydrophobic hosts.[37]	Pluth, Bergman and Raymond discuss a cation-stabilizing host:<br>“We report here a synthetic supramolecular host assembly that relies exclusively on electrostatic and hydrophobic interactions for thermodynamic stabilization of protonated substrates.“<br>“The naphthalene walls render the interior hydrophobic, whereas the tetra-anionic ligands in combination with the trivalent metal centers confer a 12^{-} overall charge to the assembly.”
Herein, I argue that an underexplored cavity type, *robust organic cages*,[38-47] are uniquely positioned to facilitate these advances.	There are many terms used to try and collect certain molecular groups into useful categories. Here, we wish to capture the class of molecules that are (i) 3D in nature (polymacrocyclic), (ii) dynamically (self)-assembled in some sense, and which are then (iii) considered robust (e.g. an amide or amine is robust, an imine is not). While the work of Andrews[38-41] and Otte[42-44] is representative, there are significant research outputs on the cusp of this classification, e.g. Davis (irreversibly assembled),[45] Liu (macrocycle only),[46] and White (sufficiently metastable.)
**Outline and overview**	
The wider history of supramolecular and cavity catalysis,[3,13,15-19,21,48,49] and catalysis using confined transition metal catalysts,[50-52] dendrimers[53] or synzymes,[54] micelles[55] or vesicles,[56] catalytic antibodies[57-59] or molecularly imprinted polymers (MIPs)[60] has been discussed elsewhere and will not be covered.	A non-exhaustive selection of significant reviews, and overviews on supramolecular catalysis.
**Cavity catalysis: current state of the art**	
**Functionalized macrocycles**	
Since Cramer’s work using cyclodextrins,[61-63]	Cramer reports decarboxylation and the fission of pyrophosphates using cyclodextrins as early as 1965.
there has been significant interest in using macrocyclic confinement to modulate reactivity.[64]	

Cyclodextrins, cucurbiturils, cavitands, and calixarenes are representative [64-70],	Selection of reviews
Since the macrocycles are generically hydrophobic on the interior, they can perform catalysis by dual-confinement of two hydrophobic substrates from water (**Figure 2A**),[71-73]	
or by binding a hydrophobic substrate and holding it close to a functional(ized) rim (e.g. cyclodextrins) that performs a reaction (**Figure 2B**).[74-79]	
These effects are driven mostly by effective concentration/molarity (i.e. proximity of reacting groups) with little,[73]	Mock reports some contribution of "Pauling catalysis" (transition state binding) in one case, but this facet is rarely discussed in macrocycle catalysis reports…
if any, transition state binding.[36,80,81]	…in contrast to dual confinement, which rests on "effective molarities".
Size-exclusion and regioselective outcomes are possible [56,82-85],	Supramolecular catalysis examples concerning selective reactions
and symmetric arrays of chiral units (like cyclodextrin) can promote enantioselectivity,[76]	In this example, the cyclodextrin acts as a "chiral ligand" for a copper-promoted reduction of ketones [76]
although turnover from augmented macrocycles is not always achieved.[26]	Scrimin reviews some fast-acting "catalysts" that are covalently deactivated by attacking a locally held substrate. Some of the "catalysts" may be reactivated (often by a slow hydrolysis or similar of the covalent macrocycle-substrate adduct).
Also in the category of (functionalized) macrocycles are large enzyme models, such as those reported by Cram,[65,86-88] Breslow,[74,75,89-92] Diederich,[93] and others,[94-96]	A rich body of literature of large molecules attempting to mimic enzymes exists.
constructed by (often laborious) linear synthesis to afford more elaborate combinations of macrocyclic cavities adorned with functional groups (**Figure 3A**).[97,98]	Nolte's polybutadiene-epoxidation system is a particularly notable effort.
mostly contributed to the view that enzymes do not work simply by bringing substrates arbitrarily close to a potentially reactive group.[99,100]	Boxer and Kirby/Hollfelder have strong discussions on this matter.
One rare but important exception is Breslow's use of two tethered cyclodextrins to locate hydrophobic esters next to a metal ion. Breslow's catalyst accelerates the hydrolysis of esters and phosphodiesters by 10^5–10^7 by electrophilic activation of ester and nucleophilic activation of water or peroxide at the metal ion [101,102].	
In enzymes, there is never just a nucleophile – there is always a metal. "oxyanion hole" or "proton wire"[103-106]	
which may explain the low activity of Cram's model serine protease in **Figure 3A**.[107]	Warshel writes: " For example, placing the focus on the general base in trypsin-inspired models (e.g., ref 2) has helped in optimizing the well understood proximity factor (see ref 5) but overlooked the key role of the oxyanion hole."
Polarization was addressed by the creative "molecular clefts" of Rebek.[108,109]	

One example featuring a pair of antipodal carboxylic acids demonstrated acetal hydrolysis catalysis (**Figure 3B**).[110]	
if catalytically useful functionality can be introduced…[to the antibodies] …model systems such as ours may become the dinosaurs of the 1990s.[108]	
Chemists thus turned toward scaffolds that were easier to access [36,111].	Lusby and Spicer introduce the benefits of self-assembly in this field, and a recent review by Lewis covers a broad range of scaffold types.
**Self-assembled containers, capsules, nanoreactors**	
In the 1990s, Rebek popularized "softball"/"tennis ball" reactors.[112-114]	Assembled from two identical 3D folds that can be "stitched" together in perfect tessellation.
These "capsules"[115-117] are two or more molecules that self-assemble via hydrogen bonds to create an internal cavity (**Figure 4A**).	
Since the parts of the capsule are dynamically assembled, substrates and products are able to enter and exit via partial disassembly of the capsule,[118]	Badjić et al discuss the mechanisms in detail.
with the only requirement being that they must fit inside.[119]	"thermodynamically controlled encapsulation process (capsule partial opening, encapsulation of guest by attractive interactions, and capsule closing)"
these capsules perform simple hydrophobic catalysis as for the macrocycles discussed in the previous section, and so remain prone to product inhibition,[113]	Rebek describes dual encapsulation of substrates.<br><br>Rebek states about one capsule: "…Although product inhibition (lack of dissociation) prevents the system from showing true catalytic behaviour…"
though not exclusively.[120]	Rebek says of an encapsulated Diels-Alder reaction: "The plot is linear over two half-life times (75% conversion), indicating that product inhibition is not a factor: true catalysis is observed"
Size- and regio- selectivity are possible.[121]	Rebek shows regioselectivity in a capsule for an azide/alkyne-click reaction. Sanders describes organised Diels-Alder reactions in porphyrin macrocycles.
Recent advances using hexameric resorcin[4]arene-based capsules[122-124]	These capsules were introduced by Atwood.
notably by Tiefenbacher,[105,125-133]	Tiefenbacher has reported extensively on the catalytic activity of these capsules.
demonstrated substrate-controlled selectivities that vary from the bulk phase due to the stabilization of cations in a size-selective space.[105,126,129,130,132,134]	A useful summary of capsule catalysis is presented here[128] but advances continue to be made.
A key advantage is that these capsules have been made on multi-hundred-gram scales and can be recycled.[132]	See abstract graphic, and within. [132]
Additionally, control of structural or bound water by the capsule[105] and properties such as a lowered pK_a inside[125]	

demonstrate a rare example of a cavity promoting catalysis via both "organization and polarization" (**Figure 4C**), including dual activation,[105] albeit with vague directionally (the substrate can be productively oriented in many conformations).	Surprisingly, although the capsule is highly symmetric (like a cube), the substrate can orientate itself such that selective polarisations can be achieved.
For instance, the window[1]-resorcin[3]arene capsule type[135] demonstrates that the symmetry and properties of traditional symmetric capsules can be modulated, although precise control over the assembly remains difficult.	The exact position of the window(s) is not well-defined, which is a common difficulty when trying to assemble lower symmetry cavities
Finally, we must mention the recently reported cavitand capsules of Gibb, who has generated large electrostatic effects by functionalizing the capsule exteriors with charged groups (**Figure 4D**).[136,137]	
**Metal-organic cages**	
The exploration of metal-organic cages (MOCs), also known as supramolecular coordination cages (SCCs), as catalysts is thriving.[22,36,138-141]	
When well-designed linkers and metals are combined, discrete cages emerge as the thermodynamic product (**Figure 5A**).[22,142-144]	
Typically, rigid linkers are required to enforce geometry, although a "weak-link" approach has been reported,[145] and flexible cages are known.[146,147]	
Application of this "directional bonding" concept[148-150] to synthesize macrocycles	…which perhaps began with Verkade.
was driven by Fujita[151–154] and others,[150,155]	
the MOCs can then be screened as catalysts, sometimes under the moniker "enzyme mimic".[22,138]	Many reports or reviews introduce the field using enzymes as an inspiration.
MOCs are typically soluble in polar organic solvents or water,[156,157] and so their dynamics can be studied using solution-phase techniques.[22]	"Host–guest interactions can be driven by the hydrophobic effect, electrostatic interactions or multiple weak interactions. Typically, host–guest complex formation can be followed by NMR spectroscopy, a valuable tool for interpretation of dynamics behind encapsulation."
As before, dual confinement/encapsulation[36] and the hydrophobic effect dominate the origin of catalytic rate enhancements.[158,159]	"In addition, some of these supramolecular complexes are highly charged and can be dispersed in aqueous media, creating a hydrophobic pocket in which to do chemistry, similar to the active site of an enzyme."
To avoid product inhibition, model reactions that increase molecularity (A → B + C) or that generate weakly interacting or less-hydrophobic[160] products have been popular, including hydrolysis, ring openings and rearrangements.[22] These reaction classes have been discussed.[24,25,140]	"The observed catalytic turnover indicated the involvement of both inclusion and exclusion steps in the catalytic cycle. The inclusion step should be driven by the efficient hydrophobic interaction between substrate 4 and cage 1 as revealed by the X-ray structure (Figure 2). The exclusion step should be driven by the reduced host–guest hydrophobic interaction due to the conversion of the hydrophobic CH2=CH– moiety into the hydrophilic CH3CO– moiety."[160]
Cavity-directed changes in ion-localisation[161,162] and pK_a are effective [37,107,163,164], and size-	

selectivity[36,165-167] and constriction (ground-state-destabilization) are also possible.[140,168-170]	
The metals can sometimes participate in redox catalysis,[171] and may be stabilized by the cage structure.[160,172-174]	Fujita: "Since the Pd(II) component, (tmed)Pd(NO_3)$_2$, 10 hardly showed the catalytic activity (only 2% yield), the present reaction was obviously catalyzed by the Pd(II)-containing cage 1 itself. Interestingly, Pd(0) species that should be formed in the Wacker-type oxidation seemed to be aerobically reoxidized into Pd(II) without using reoxidizing coreagent such as a Cu(II) salt." [160]<br><br>"Self-assembled nanocage 1 was found to promote the aerobic, aqueous oxidation of styrene and its derivatives with the aid of (en)Pd(NO_3)$_2$. Obviously, the reaction was promoted by a double catalysis system: i.e., cage 1 acted as a reverse phasetransfer catalyst, whereas (en)Pd_2^+ as an oxidation catalyst"[172]
The organic part of the MOC has also been levied as a hydrogen-bond donor to activate an electrophile.[175]	"The **P** was employed as an effective hydrogen-bond-donor catalyst for Michael reactions of a series of water-insoluble nitro-olefins in an aqueous medium."
In terms of *polarization*, since cages are invariably charged,[36]	"Coordination cages have also emerged as promising successors to the early covalent receptors. These cages are not only synthetically more accessible than both covalent cages and even hydrogen bond assembled systems, they are also invariably charged."
A key example uses the highly successful Raymond gallium-based cages, exploited by Raymond, Bergman and Toste,[21,37,107,155,165,168,169,176-179]	Arguably the most successful MOC catalysis system known to date.
catalysis is again predominantly entropically driven inside the cavity, for instance by constriction (*organization*).[36,180,181]	Di Stefano discusses rates of the 3-aza-Cope rearrangement catalyzed by the gallium cage and finds marginal enthalpic stabilizations; the reaction is entropically driven.
Likewise, the cationic cages of Fujita[151,154,160,170,172,182-189] can stabilize anionic species.[184]	
The work of Lusby [166,190-196] is notable for using the metals in the cage framework to polarize adjacent aryl-hydrogen bonds in the ligands by enough to coordinate and activate substrates (**Figure 5E**).[190]	Using cage types first reported by Richard Hooley
A valuable analysis of catalytic modes has been performed.[140]	
Because MOCs are simple to assemble, modular functionalization of the periphery, sometimes internally projected [164,197,198],	
If it is easier for a cage to flex[146]	
The trade-off of rapid synthesis of MOCs by self-assembly is that they can lack robustness, and so catalysis conditions have to be mild enough to avoid cage disassembly,[22] although	

compartmentalization of contrasting reactivities is possible to avoid such incompatibilities.[189,199-201]	
Likewise, the lability of metal-ligand dative bonds can make post-assembly modifications of MOCs challenging,[202,203]	
reactions that transform the ligands can result in changes in cage topology distribution.[204]	For instance, interconversion between tetrahedra and lantern type structures.
Self-assembly also prescribes limitations on the symmetry of cages.[36]	Lusby writes: “For instance, they often lack the robustness of systems that are<br>prepared using covalent chemistry. It is also difficult to generate assemblies<br>that contain any appreciable levels of asymmetry.”
Notably, Clever has recently reported the first Pd_2(ABCD) MOC,[205] which may signal the possibility of using low-symmetry cavities[206-215] for catalysis.	
dynamic MOCs, which show promise in systems chemistry.[200]	
“Switchable” metal organic cages[212] use a stimulus like light to change ligand geometries.	
though where geometric changes are tolerated within the original structure the stimuli can trigger guest- release [216]and even switch catalysis on and off [196].	
MOCs containing peptides in the edge pieces also look promising to direct catalysis.[217]	
**Extended frameworks**	
Metal Organic Frameworks (MOFs)[218,219]	
and Covalent Organic Frameworks (COFs)[220-224]	
The catalytic properties of MOFs compared to MOCs have been discussed.[22]	
many just operate as solid-supported versions of existing catalytic motifs.[225,226]	“This design principle opens a chance to explore COFs as heterogeneous catalysts to promote chemical reactions”<br><br>“In spite of the great potential for MOFs in heterogeneous catalysis, very few of them have been explored for catalytic applications. This is in part due to the relative thermal and hydrolytic instability of MOFs as compared to zeolites.” (2009)
so the resulting cavities are often quite large and channels can deform if solvent is absent.[227]	“Framework stability and accessibility of the open channels to reagents are then addressed.”<br><br>“ Such large open channels are essential for asymmetric catalytic reactions because of the need to transport organic substrates and products that are typically quite sizeable.”<br><br>“Chiral MOFs with large open channels (typically larger than 1.5 nm) are desired due to the large size of prochiral substrates and the resulting chiral products. Unfortunately, MOFs with large open channels tend to undergo significant

	framework distortion upon the removal of solvent molecules”
Lattices can also contain defects, which may affect catalytic activity unpredictably.[228]	“…reported that a direct correlation exists between the amount of missing linker defects and the catalytic activity of UiO-66 materials for the acid-catalysed esterification of levulinic acid with various alcohols…”<br><br>“B.3. Catalysis on lattice defects”
dense arrays of aligned C–H bonds can provide CH–pi interactions in Diels-Alder catalysis.[229]	“The acceleration of the Diels–Alder reactions by the Py–An COF catalyst is likely related to the enhanced reactant concentration within the pores of the COFs and C–H···π interactions between the π-walls of COFs with 9-hydroxymethylanthracene… … The C–H···π interactions between the pore walls and 9-hydroxymethylanthracene reduced the entropy loss and activation energy of the reactions.”
crucial techniques like solution phase-NMR are rarely useful.[22]	“In future, characterization of transient intermediates with conventional solution-based techniques such as time-resolved UV–vis spectroscopy would also be desirable. This is, however, complicated by the heterogeneous nature of MOFs.”<br><br>“Since MOFs are heterogeneous materials, investigation of processes has mainly focused on solid-state techniques such as X-ray diffraction (XRD) and X-ray absorption spectroscopy (XAS).”
Enzyme encapsulation,[230] and electro- and photo-catalysis are known,[228]	
chiral and low symmetry MOFs are an exciting avenue, although the synthesis and characterization (particularly crystallization) of low-symmetry structures remains challenging.[227,231,232]	Kim reports an enantioselective MOF catalyst.<br><br>“However, compared with the achiral MOFs, the number of CMOFs is very small (Fig. 1). The reason is that the CMOFs with low symmetry are difficult to crystallize and structural characterization is also challenging, and the most important drawback is that chiral reagents are usually expensive and difficult to obtain.”[232]
Likewise, COFs hosting chiral organocatalysts are known (**Figure 6B**).[226,233]	
and multiple reactive sites can be introduced into a single rigid framework,[234]	“Unlike in solutions, acid and basic sites can be easily installed and immobilized in the MOF lattice. Intimate contact between two sites is avoided by a lack of mobility and spatial separation between the acid and basic centers. In this way, the two types of sites can simultaneously coexist and cooperate with the reaction mechanism in such a way that the reaction proceeds in a solid more efficiently than expected considering the weak acid/base strength of the sites” [234]

**Covalent organic cages**	
Covalent Organic Cages[235] are any discrete assembly, at least bicyclic in connectivity, whose minimal structure is comprised of covalent bonds	"Cage" chemistry is full of niche terms for 3D architectures. In my view, the signifier "covalency" distinguishes cages from capsules, "organic" indicates a lack of structurally essential metals, and "bicyclic" distinguishes them from macrocycles, at least formally. This structural distinction says little about properties, since nanohoops/nanobelts/nanotubes etc might present larger or more rigid 3D cavities than many "cages".
Organic cages have historically been synthesized using irreversible bond formation [236-248], sometimes with a template [45,249,250].	Irreversible bond formation is still a popular assembly approach, in part because it allows more strained products to be accessed since irreversible reaction driving forces can be harnessed. Typically, however, the lack of reversibility results in competing oligomerisation and lower yields. Templates can help overcome strain and increase yields.
Following the popularization of Dynamic Covalent Chemistry in the 1990s,[251-255]	
macrocycle and cage synthesis using reversible reactions [256-259] like imine formation (**Figure 7A**) have led to advances in the synthesis of COFs[223,224] and discrete organic cages [260-284]	We do not provide an exhaustive list of cage examples; a more complete review is given by Martínez-Máñez, Martí-Centelles et al.[235]
notably Porous Organic Cages (POCs) since 2008 [285-289], by dynamic covalent "self-assembly" (**Figure 7B**) [258].	This development has been spearheaded by Mastalerz and Cooper (particularly in applying them to gas adsorption). Similar assembly strategies were pursued at the same time by Gawronski.
In these cases, although covalent bonds are formed, bond formation is still self-directing and self-correcting, and the term "self-assembly"[290-292] often used.[286,293-296]	Lindsey gives an early definition of self-assembly.<br><br>The term self-assembly has been used frequently for covalently assembled structures, as long as there is a reversible mechanism that in principle allows the thermodynamic product to prevail.
Products can be thermodynamic or kinetic, but are often isolated by precipitation due to low solubility,[297] though many have useful solubility.[288,298]	"Porous organic molecular materials are a subclass of porous solids that are defined by their modular, molecular structures, and the absence of extended covalent or coordination bonding in the solid-state. As a result, porous molecular materials are soluble"
POCs have little internal functionality in their cavities, they have found far greater utility as porous solids (e.g. for gas uptake or separations) than as catalysts.[287,288,298-314]	
Catalysis with organic cages has only emerged in the past few years,[20,70,235,315] and true organocatalysis is exceedingly rare.[316]	Examples of recent reviews and textbooks that do not mention organic cages in the context of catalysis.<br>Mukherjee has recently reviewed credible cases.<br>One example of organic cages performing organocatalysis is Yuan's POC which has proline residues on the framework. This is no doubt organocatalysis, but the role of the framework is unclear i.e. it does not appear to work like an enzyme, by using the environment to modulate

	reactivity (it is unclear how catalysis can be said to emerge from the environment).[316]
Instead, catalytic systems tend to be composed of cavities that increase substrate solubility,[317] or host nanoparticles,[318-326] metals,[44,327,328], photoactive groups,[329-331] superbases,[332-336] or non-specific arrays of hydrogen bond donors[337]/acceptors.[338]	
Advances in post-assembly modifications[339] have recently allowed stable organic cages featuring endohedral (internally directed) functionality,[340,341] or metals,[342-345] which show early promise for low-symmetry cavities with catalytic potential.[42-44,340]	
Notably, Otte has used semi-stepwise self-assembly … to access a robust organic cage … which can act as an oxidation catalyst (**Figure 7C**).[44]	
**Perspective: on new directions for organic cages**	
**Functionalizable, stable, soluble organic cages as cavity catalysts**	
Surprisingly, despite the fact enzymes are predominantly organic molecules performing organocatalysis,[100]	
cavity-based organocatalysis remains under-represented in the supramolecular literature.[4,70]	A recent chapter entitled "Supramolecular Organocatalysis" did not mention a single organic cage.[4]
My laboratory has therefore attempted to understand the reason for this deficit, and to identify solutions.[38-41]	
Firstly, cavities containing oriented functional organic groups (like those that participate in enzyme active sites) remain conspicuously rare.[340]	Otte has reported a macrocycle with frustrated functionality. "To date, examples of shape-persistent macrocyles offering a heterosequenced, less symmetrical interior are rare."
Indeed, a recent perspective[21] identified two approaches to site-selectivity in supramolecular host catalysis – (i) using the host as a "protecting group" to direct reactivity external to the host,[187,346] and (ii) confinement of a transition metal catalyst to take advantage of the restricted environment of the host[51,52]	"The supramolecular protecting group approach enables high levels of selectivity across different host-guest platforms and organic reactions, but its application requires quantitative formation of the host-guest complex and super-stoichiometric concentrations of host, which can limit its scope and scalability. An alternative approach that promotes high selectivity at catalytic loading of host involves anchoring a reactive metal catalyst internally, thereby restricting the size and conformations of substrates that can co-encapsulate"
– neither of which resembles the enzyme-like possibility of a true active site (binding a substrate in an orientation that directs internally-catalyzed reactivity).[347,348]	"In Nature the relevant enzymatic reactions involve oxidation by metalloporphyrins, with reversible enzyme binding of the substrate in such a geometry that specific substrate positions are within reach of the oxygen atom on the metal." [348]
The examples of cavities with functionality discussed above, from Cram's "full serine	Discussion of organisation in supramolecular chemistry.[180]

protease model"[87], to MOCs with flexible peripheral groups,[349] to frameworks with internal proline organocatalysts[222,234] all suffer from the same limitation: they all fail to rigidly organize sufficient bifunctional groups to obtain clear transition state binding – hallmark of enzymes and organocatalysts [107,180].	
We quickly identified the triptycenyl-based imine cages of Mastalerz[301] as a strong starting point	
the privileged triptycene motifs…for which extensive synthetic development is known;[350-355]	There are few organic motifs that provide the precise combination of vectors offered by triptycenes.
they are the lowest possible symmetry polymacrocyclic structure (removing one edge piece will result in a macrocycle),[356,357]	
Otte has levied similar techniques to generate soluble amine-based organic cages with the same symmetry possibilities, using the three edge pieces to provide internal vectors to generate "catalytic triad" mimics.[42-44]	
our chosen cage frameworks,[300,302] at least, were poorly soluble,[297]	
and required development to exploit them in the solution state (**Figure 8A**).[38]	
To capture both stability and solubility, we turned to Mastalerz's post-functionalization chemistry,[286,300,304,306,358.-362]	
an approach gaining popularity.[46,262]	
in which imines are oxidized by a Pinnick oxidation to amides.[286,360,363,364]	Mastalerz has applied adapted Pinnick conditions to cages, which were previously used in COF chemistry: [286,360,363,364]
Importantly, we were able to adapt this chemistry to work *in situ*, allowing soluble metastable imine frameworks to be trapped as amides.[38]	
The adapted cages were soluble, and were now stable enough to be purified by gel-permeation (size-exclusion) chromatography (useful when precipitation is not possible), which is typically not possible for imine[314] or metal-coordination cages.[156,202,365]	
allowing access to the key acid-functionalized cages[38]	
Otte has employed reductive amination to stabilize imine cages, and the resulting amine cages gain solubility from increased flexibility at the cost of some structural rigidity.[42-44]	
**C1-C13** (**Figure 9A**).[39,40]	
population consisting of perhaps two major conformers (**C5**, **C9**), with 3-4 minor contributors (298 K, THF),[39,40]	
introducing functionality which favors particular amide orientations.[39]	
For instance, hexapyridine cage **2** (**Figure 8B**) shows exclusive occupation of **C13** (all carbonyls	

out) due to six favorable $NH_{amide} \rightarrow N_{pyr}$ interactions,[41]	
a low-symmetry (C_S) conformation consistent with **C5** (**Figure 8A**).[39]	
While observations of symmetry-lowering are commonplace in controlled environments,[47,264,293,335,366-369]	Examples of cages with amides and imines where the macrocyclic environment leads to metastable conformers with low symmetry
we believe there to be wide-ranging potential across low-symmetry cavity research [205,208,309,366,370–374]	
We were also able to statistically access a cage with a single internal carboxylic acid group and purify the cage by size-exclusion (GPC) due to the cage robustness.[41]	Imines tend to equilibrate during GPC purification, leading to broad streaking as various sizes/oligomers form. Amide cages are robust and maintain bond integrity and therefore a narrow size.
Use of bulky internal groups can statistically bias cage self-assembly of mixed groups, a strategy previously reported.[375]	
studied in the solution state as hosts for diamines (**Figure 8B**),[38,39] guests which would ostensibly degrade host imine cages or metal-organic cages.[345]<br>Indeed, imines cages are rarely viable as solution-phase hosts.[263,342,343,345]	Zonta has designed imine cages that can disassemble when the guest size is too big.
Further, without many-electron metals to model,[208,376] high-throughput calculations of organic cages are facile,[366,377-385] and do not require metal parameterization.[140,386,387]	
As well as tolerating higher temperatures and more reactive reagents ("cofactors"), they could perform non-biotic reactions, and even have potential for the design of novel active sites that could be transposed into new designer enzymes for optimization.[388-391]	Enzymes can be tuned by directed evolution, but only if there is some activity to use as a selection pressure. Introducing even poor synthetic active sites into enzymes may be sufficient to tune towards higher activity.
… too flexible or too rigid to mimic enzymes. This is not the place for a detailed discussion on the nuances of these vague terms in enzymology,[392-394]	
This "macro-flexibility" is more akin to large enzyme-like movements that might permit tuning of several consecutive catalytic steps,[395] induced-fit binding, product release, allostery,[396,397] or signalling	
The ability to tune catalytic activity by tuning the cage rigidity, conformation, or dynamics is certainly an advantage rather than a liability.[395,398-402]	
It is perhaps unsurprising that the balance of rigidity and flexibility[403,404] contributed by the amide links in proteins seems to be well-reproduced in our amide-linked cages.	"However, in contrast to enzymes, which bind the transition state by multiple contacts *via* an induced fit mechanism, many of the low molecular weight model systems exhibit only minor catalytic activity due to lack of catalytic groups and too much rigidity or flexibility."

	"Enzymes exhibit a certain degree of flexibility and can bind the substrate and transition state by an induced fit mechanism, whereas the biomimetic compounds are often too rigid or too flexible."
**Automation and calculation in cavity synthesis and study**	
The computational discovery of new materials has advanced in recent years with increased computational power.[405]	Jelfs et al have discussed new materials discovery. The state-of-the-art remains limited: "Given the significant efforts in computational materials discovery, including a large field in high-throughput computational screening, it is not unreasonable to ask why are there, by comparison, relatively few experimentally realized materials that originated from computational predictions?"
Imine-based porous organic cages have been a popular choice for study,[377,380,406-408] as have MOCs.[208,376,386]	
Much focus remains on the prediction (and automation)[409] of the formation of cavities by probing combinations of (e.g.) amines/aldehydes or metals/ligands to identify structures with clear thermodynamic minima.[410]	
within a "near infinite design space".[376]	"As metal–organic cages may be comprised from nearly any combination of organic and metal-containing components, cages can form with diverse shapes and sizes, allowing for tuning toward targeted properties. Therefore, their near-infinite design space is almost impossible to explore through experimentation alone and computational design can play a crucial role in exploring new systems."
Further, by definition, screening for particularly stable assemblies[405,411] screens out cages with the taut, dynamic properties found in biological systems.[100,146,395,401,412]	"…Jansen and Schön argued that rational development of materials is a fallacy, because the thermodynamic viability of a material must be considered as part of the discovery process. They argue it is irrelevant if a hypothetical material has optimal properties if it is thermodynamically (or kinetically) unstable and as such could never be synthesized or operate in a given application. Instead, they put forward the necessity of screening processes that first assess the thermodynamic viability of hypothetical material candidates, only taking forward to property screening those materials that are energetically viable. Despite advances in hardware and computation software, computational stability assessment for hypothetical materials is extremely challenging, often relying on calculations of formation enthalpies with electronic structure methods. The energy landscape for a single material alone is often complex. Thus, from an inverse design perspective, selecting the optimal material candidate from a material space is highly computationally intensive and challenging"

	Lewis describes the roles that flexible cages might play.[146] Enzyme dynamics, of course, need dynamical systems, which must entail some form of flexibility. [100,395,401,412]
Instead, new receptors [413] and catalysts [414–416] will likely arise from improvements in computational rationalization of experimental data [415,417],	Research programmes investigating receptor and catalyst design.
. In turn, this process will be enabled by improving automated experimental screening of existing cavities for new activity (sensing, catalysis) [418].	Even the state of the art in automated chemistry has yet to provide meaningful discoveries.
The lack of internal functionality in cavities doesn't just reduce the possibility of larger *polarization* contributions to catalysis, it also makes property prediction difficult[419]	
Materials with more precise substrate and transition state binding modes,[41,105] such as those found in robust organic cages, can therefore be readily studied, understood, and improved.	
**Conclusions and final perspective**	
This follows from the requirement for differential transition state binding over substrate binding for catalysis,[100] and requires cavities that *polarize* in addition to *organizing*, that is, an *organized polarization*.[420]	
Quantifying patterns found in (automated) screening will be vital to ensure that unintuitive geometric[39,205,207,211,366,367] and thermodynamic rules inform sequential iteration,[96,421,422]	Webb and Wilcox discuss carefully organised polar functionality in this review.
since the supramolecular landscape is vast, and highly successful systems still tend to be those patiently developed[45,248,423,424] (as nature has) rather than discovered in a few simple reactions.	
One is reminded of Rebek's trite observation that host systems are frequently chosen on the basis of simple synthetic accessibility, briefly screened for activity, and "…the word *design* … much over-used in the publication."[425]	
In terms of application focus, site-selectivity in polyfunctionalized (e.g. bio-derived) materials[426-428] is of increasing importance	
The need for *polarization* may be circumvented by incorporating photocatalysts into cages,[183,429] which are also likely to provide novel site-selective reactions,[21,185,430-433]. Cage structure may also activate photocatalysts[434] or help restrict detrimental photocatalyst deactivation reactions [435].	
metastable cavities are less likely to tolerate the structural strain that switching can induce.[345,436,437]	
we also anticipate that structurally-tailored robust organic cage structures will finally allow access to more relevant enzyme mimics, organocatalytic	

motifs, and functional triads in precise-enough ways to permit a new wave of enzyme model studies, this time to reveal details of electric field catalysis [99,417,438-440] and the elusive roles of enzyme dynamics.[395,401,441]	

References

(1) Wolfenden, R.; Snider, M. J. *Acc. Chem. Res.* **2001**, *34*, 938–945. doi:10.1021/AR000058I

(2) Beddoe, R. H.; Andrews, K. G.; Magné, V.; Cuthbertson, J. D.; Saska, J.; Shannon-Little, A. L.; Shanahan, S. E.; Sneddon, H. F.; Denton, R. M. *Science* **2019**, *365*, 910–914. doi:10.1126/SCIENCE.AAX3353

(3) Mitschke, B.; Turberg, M.; List, B. *Chem* **2020**, *6*, 2515–2532. doi:10.1016/J.CHEMPR.2020.09.007

(4) Woods, P. A.; Smith, A. D. Supramolecular Organocatalysis. In *Supramolecular Chemistry*; Wiley, 2012. doi:10.1002/9780470661345.smc156

(5) Ma, J.-A.; Cahard, D.; Cahard, D.; Ma, J.-A. *Angew. Chem. Int. Ed.* **2004**, *43*, 4566–4583. doi:10.1002/ANIE.200300635

(6) Sawamura, M.; Ito, Y. *Chem. Rev.* **1992**, *92*, 857–871. doi:10.1021/CR00013A005

(7) Steinhagen, H.; Helmchen, G. *Angew. Chem. Int. Ed.* **1996**, *35*, 2339–2342. doi:10.1002/ANIE.199623391

(8) Shibasaki, M.; Sasai, H.; Arai, T. *Angew. Chem. Int. Ed.* **1997**, *36*, 1236–1256. doi:10.1002/ANIE.199712361

(9) Van Den Beuken, E. K.; Feringa, B. L. *Tetrahedron* **1998**, *54*, 12985–13011. doi:10.1016/S0040-4020(98)00319-6

(10) Rowlands, G. J. *Tetrahedron* **2001**, *57*, 1865–1882. doi:10.1016/S0040-4020(01)00057-6

(11) Gröger, H. *Chem. Eur. J.* **2001**, *7*, 5246–5251. doi:10.1002/1521-3765(20011217)7:24<5246::aid-chem5246>3.0.co;2-o

(12) Shibasaki, M.; Yoshikawa, N. *Chem. Rev.* **2002**, *102*, 2187–2209. doi:10.1021/CR010297Z

(13) Lehn, J. M. *Proc. Natl. Acad. Sci.* **2002**, *99*, 4763–4768. doi:10.1073/PNAS.072065599

(14) Antipin, I. S.; Alfimov, M. V.; Arslanov, V. V.; Burilov, V. A.; Vatsadze, S. Z.; Voloshin, Y. Z.; Volcho, K. P.; Gorbatchuk, V. V.; Gorbunova, Y. G.; Gromov, S. P.; Dudkin, S. V.; Zaitsev, S. Yu.; Zakharova, L. Ya.; Ziganshin, M. A.; Zolotukhina, A. V.; Kalinina, M. A.; Karakhanov, E. A.; Kashapov, R. R.; Koifman, O. I.; Konovalov, A. I.; Korenev, V. S.; Maksimov, A. L.; Mamardashvili, N. Zh.; Mamardashvili, G. M.; Martynov, A. G.; Mustafina, A. R.; Nugmanov, R. I.; Ovsyannikov, A. S.; Padnya, P. L.; Potapov, A. S.; Selektor, S. L.; Sokolov, M. N.; Solovieva, S. E.; Stoikov, I. I.; Stuzhin, P. A.; Suslov, E. V.; Ushakov, E. N.; Fedin, V. P.; Fedorenko, S. V.; Fedorova, O. A.; Fedorov, Y. V.; Chvalun, S. N.; Tsivadze, A. Yu.; Shtykov, S. N.; Shurpik, D. N.; Shcherbina, M. A.; Yakimova, L. S. ***Russ. Chem. Rev.*** **2021**, *90*, 895–1107. doi:10.1070/RCR5011/XML

(15) Chai, Y.; Dai, W.; Wu, G.; Guan, N.; Li, L. *Acc. Chem. Res.* **2021**, *54*, 2894–2904. doi:10.1021/ACS.ACCOUNTS.1C00274

(16) Van Leeuwen, P. W. N. M.; Raynal, M. *Supramolecular Catalysis: New Directions and Developments* **2021**, 1–646. doi:10.1002/9783527832033

(17) Van Leeuwen, P. W. N. M. *Supramolecular Catalysis* **2008**, 1–303. doi:10.1002/9783527621781

(18) Raynal, M.; Ballester, P.; Vidal-Ferran, A.; Van Leeuwen, P. W. N. M. *Chem. Soc. Rev.* **2014**, *43*, 1734–1787. doi:10.1039/C3CS60037H

(19) Raynal, M.; Ballester, P.; Vidal-Ferran, A.; Van Leeuwen, P. W. N. M. *Chem. Soc. Rev.* **2014**, *43*, 1660–1733. doi:10.1039/C3CS60027K

(20) Pappalardo, A.; Puglisi, R.; Sfrazzetto, G. T. *Catalysts* **2019**, *9*, 630. doi:10.3390/catal9070630

(21) Morimoto, M.; Bierschenk, S. M.; Xia, K. T.; Bergman, R. G.; Raymond, K. N.; Toste, F. D. *Nat. Catal.* **2020**, *3*, 969–984. doi:10.1038/s41929-020-00528-3

(22) Pullen, S.; Clever, G. H. *Monographs in Supramolecular Chemistry* **2021**, *2021-January*, 247–281. doi:10.1039/9781788019705-00247

(23) Basilio, N.; García-Río, L.; Moreira, J. A.; Pessêgo, M. *J. Org. Chem.* **2010**, *75*, 848–855. doi:10.1021/JO902398Z
(24) Syntrivanis, L.-D.; Tiefenbacher, K. *Angew. Chem. Int. Ed.* **2024**, e202412622. doi:10.1002/ANIE.202412622
(25) Sanders, J. K. M. *Chem. Eur. J* **1998**, *4*, 1378–1383. doi:10.1002/(SICI)1521-3765(19980807)4:8<1378::AID-CHEM1378>3.0.CO;2-3
(26) Lyu, Y.; Scrimin, P. *ACS Catal.* **2021**, *11*, 11501–11509. doi:10.1021/acscatal.1c01219
(27) Jiao, J.; Li, Z.; Qiao, Z.; Li, X.; Liu, Y.; Dong, J.; Jiang, J.; Cui, Y. *Nat. Commun.* **2018**, *9*, 4423. doi:10.1038/s41467-018-06872-0
(28) Chattaraj, P. K.; Sarkar, U. *J. Phys. Chem. A* **2003**, *107*, 4877–4882. doi:10.1021/JP034321J
(29) Márquez, F.; García, H.; Palomares, E.; Fernández, L.; Corma, A. *J. Am. Chem. Soc.* **2000**, *122*, 6520–6521. doi:10.1021/JA0003066
(30) Borgoo, A.; Tozer, D. J.; Geerlings, P.; De Proft, F. *Phys. Chem. Chem. Phys.* **2008**, *10*, 1406–1410. doi:10.1039/B716727J
(31) Schoonheydt, R. A.; Weckhuysen, B. M.; Meurig Thomas, J.; Carlos Hernandez-Garrido, J.; Raja, R.; Bell, R. G.; Chem Chem Phys, P. *Phys. Chem. Chem. Phys.* **2009**, *11*, 2862–2868. doi:10.1039/B820114E
(32) Corma, A.; García, H.; Sastre, G.; Viruela, P. M. *J. Phys. Chem. B* **1997**, *101*, 4575–4582. doi:10.1021/JP9622593
(33) Warshel, A.; Sharma, P. K.; Kato, M.; Xiang, Y.; Liu, H.; Olsson, M. H. M. *Chem. Rev*. **2006**, *106*, 3210–3235. doi:10.1021/cr0503106
(34) Warshel, A. *Proc. Natl. Acad. Sci.* **1978**, *75*, 5250–5254. doi:10.1073/PNAS.75.11.5250
(35) Warshel, A. *Acc. Chem. Res.* **1981**, *14*, 284–290. doi:10.1021/AR00069A004
(36) Spicer, R. L.; Lusby, P. J. Chapter 2: Catalytic Strategies within the Confined Spaces of Coordination Cages. In *Monographs in Supramolecular Chemistry*; Royal Society of Chemistry, 2021; Vol. 2021-Janua, pp 29–69. doi:10.1039/9781788019705-00029
(37) Pluth, M. D.; Bergman, R. G.; Raymond, K. H. *Science* **2007**, *316*, 85–88. doi:10.1126/SCIENCE.1138748
(38) Andrews, K. G.; Christensen, K. E. *Chem. Eur. J.* **2023**, e202300063. doi:10.1002/chem.202300063
(39) Andrews, K. G.; Horton, P. N.; Coles, S. J. *Chem. Sci.* **2024**, *15*, 6536–6543. doi:10.1039/D4SC00889H
(40) Shields, C. E.; Fellowes, T.; Slater, A. G.; Cooper, A.; Andrews, K. G.; Szczypiński, F. T. *Chem. Commun.* **2024**, *60*, 6023–6026. doi:10.1039/D4CC01407C
(41) Andrews, K. G.; Piskorz, T. K.; Horton, P. N.; Coles, S. J. *J. Am. Chem. Soc.* **2024**, *146*, 17887–17897. doi:10.1021/jacs.4c03560
(42) Bete, S. C.; Würtele, C.; Otte, M. *Chem. Commun.* **2019**, *55*, 4427–4430. doi:10.1039/C9CC00437H
(43) Otte, M.; Lutz, M.; Klein Gebbink, R. J. M. *Eur. J. Org. Chem.* **2017**, *2017*, 1657–1661. doi:10.1002/EJOC.201700106
(44) Bete, S. C.; May, L. K.; Woite, P.; Roemelt, M.; Otte, M. *Angew. Chem. Int. Ed.* **2022**, *61*, e202206120. doi:10.1002/ANIE.202206120
(45) Tromans, R. A.; Carter, T. S.; Chabanne, L.; Crump, M. P.; Li, H.; Matlock, J. V.; Orchard, M. G.; Davis, A. P. *Nat. Chem.* **2019**, *11*, 52–56. doi:10.1038/s41557-018-0155-z
(46) Zhai, C.; Xu, C.; Cui, Y.; Wojtas, L.; Liu, W. *Chem. Eur. J.* **2023**, e202300524. doi:10.1002/CHEM.202300524
(47) Foyle, É. M.; Goodwin, R. J.; Cox, C. J. T.; Smith, B. R.; Colebatch, A. L.; White, N. G. *J. Am. Chem. Soc.* **2024**. doi:10.1021/JACS.4C09930
(48) Lehn, J. -M. *Angew. Chem. Int. Ed.* **1988**, *27*, 89–112. doi:10.1002/ANIE.198800891
(49) Otte, M. *ACS Catal.* **2016**, *6*, 6491–6510. doi:10.1021/ACSCATAL.6B01776
(50) Bellini, R.; Chikkali, S. H.; Berthon-Gelloz, G.; Reek, J. N. H. *Angew. Chem. Int. Ed.* **2011**, *50*, 7342–7345. doi:10.1002/anie.201101653
(51) Nurttila, S. S.; Brenner, W.; Mosquera, J.; van Vliet, K. M.; Nitschke, J. R.; Reek, J. N. H. *Chem. Eur. J.* **2019**, *25*, 609–620. doi:10.1002/CHEM.201804333
(52) Jongkind, L. J.; Caumes, X.; Hartendorp, A. P. T.; Reek, J. N. H. *Acc. Chem. Res.* **2018**, *51*, 2115-2128. doi:10.1021/acs.accounts.8b00345
(53) Astruc, D.; Chardac, F. *Chem. Rev.* **2001**, *101*, 2991-3024. doi:10.1021/cr010323t

(54) Klotz, I. M.; Royer, G. P.; Scarpa, I. S. *Proc. Natl. Acad. Sci.* **1971**, *68*, 263-264. doi:10.1073/pnas.68.2.263
(55) Dwars, T.; Paetzold, E.; Oehme, G. *Angew. Chem. Int. Ed*. **2005**, *44*, 7174-7199. doi:10.1002/anie.200501365
(56) Vriezema, D. M.; Aragonès, M. C.; Elemans, J. A. A. W.; Cornelissen, J. J. L. M.; Rowan, A. E.; Nolte, R. J. M. Self-Assembled Nanoreactors. *Chem. Rev.* **2005**, *105*, 1445-1490. doi:10.1021/cr0300688
(57) Janda, K. D.; Schloeder, D.; Benkovic, S. J.; Lerner, R. A. *Science* **1988**, *241*, 1188–1191. doi:10.1126/science.3413482
(58) Baldwin, E.; Schultz, P. G. *Science* **1989**, *245*, 1104–1107. doi:10.1126/science.2672338
(59) Mader, M. M.; Bartlett, P. A. *Chem. Rev.* **1997**, *97*, 1281–1301. doi:10.1021/CR960435Y
(60) Wulff, G.; Liu, J. *Acc. Chem. Res.* **2012**, *45*, 239–247. doi:10.1021/AR200146M
(61) Hennrich, N.; Cramer, F. *J. Am. Chem. Soc.* **1965**, *87*, 1121–1126. doi:10.1021/JA01083A032
(62) Cramer, F.; Dietsche, W. *Chem. Ber.* **1959**, *92*, 1739–1747. doi:10.1002/CBER.19590920804
(63) Cramer, F.; Kampe, W. *J. Am. Chem. Soc.* **1965**, *87*, 1115–1120. doi:10.1021/JA01083A031
(64) Sadjadi, S. *Organic Nanoreactors: From Molecular to Supramolecular Organic Compounds*; Academic Press, 2016, pp 1–569. doi:10.1016/C2014-0-00516-3
(65) Cram, D. J. *Nature* **1992**, *356*, 29–36. doi:10.1038/356029a0
(66) Crini, G. *Chem. Rev.* **2014**, *114*, 10940–10975. doi:10.1021/CR500081P
(67) Assaf, K. I.; Nau, W. M. *Chem. Soc. Rev.* **2014**, *44*, 394–418. doi:10.1039/C4CS00273C
(68) Zhang, D.; Martinez, A.; Dutasta, J. P. *Chem. Rev.* **2017**, *117*, 4900–4942. doi:10.1021/acs.chemrev.6b00847
(69) Sachdeva, G.; Vaya, D.; Srivastava, C. M.; Kumar, A.; Rawat, V.; Singh, M.; Verma, M.; Rawat, P.; Rao, G. K. *Coord. Chem. Rev.* **2022**, *472*, 214791. doi:10.1016/J.CCR.2022.214791
(70) Breiner, B.; Nitschke, J. R. Reactivity in Nanoscale Vessels. In *Supramolecular Chemistry*; Wiley, 2012. doi:10.1002/9780470661345.smc163
(71) Breslow, R.; Campbell, P. *Bioorg. Chem.* **1971**, *1*, 140–156. doi:10.1016/0045-2068(71)90012-5
(72) Rideout, D. C.; Breslow, R. *J. Am. Chem. Soc.* **1980**, *102*, 7816–7817. doi:10.1021/JA00546A048
(73) Mock, W. L.; Irra, T. A.; Wepsiec, J. P.; Manimaran, T. L. *J. Org. Chem.* **1983**, *48*, 3619–3620. doi:10.1021/JO00168A070
(74) Breslow, R.; Dong, S. D. *Chem. Rev.* **1998**, *98*, 1997–2011. doi:10.1021/cr970011j
(75) Breslow, R.; Overman, L. E. *J. Am. Chem. Soc.* **1970**, *92*, 1075–1077. doi:10.1021/ja00707a062
(76) Xu, G.; Leloux, S.; Zhang, P.; Meijide Suárez, J.; Zhang, Y.; Derat, E.; Ménand, M.; Bistri-Aslanoff, O.; Roland, S.; Leyssens, T.; Riant, O.; Sollogoub, M. *Angew. Chem. Int. Ed.* **2020**, *59*, 7591–7597. doi:10.1002/ANIE.202001733
(77) Roland, S.; Suarez, J. M.; Sollogoub, M. *Chem. Eur. J.* **2018**, *24*, 12464–12473. doi:10.1002/CHEM.201801278
(78) Komiyama, M.; Bender, M. L. *J. Am. Chem. Soc.* **1977**, *99*, 8021–8024. doi:10.1021/JA00466A040
(79) Rebilly, J. N.; Colasson, B.; Bistri, O.; Over, D.; Reinaud, O. *Chem. Soc. Rev.* **2014**, *44*, 467–489. doi:10.1039/C4CS00211C
(80) Cacciapaglia, R.; Di Stefano, S.; Mandolini, L. *Acc. Chem. Res.* **2004**, *37*, 113–122. doi:10.1021/AR020076V
(81) Kirby, A. J. *Adv. Phys. Org. Chem.* **1980**, *17*, 183–278. doi:10.1016/S0065-3160(08)60129-X
(82) Nakash, M.; Clyde-Watson, Z.; Feeder, N.; Davies, J. E.; Teat, S. J.; Sanders, J. K. M. *J. Am. Chem. Soc.* **2000**, *122*, 5286–5293. doi:10.1021/JA9922227
(83) Walter, C. J.; Anderson, H. L.; Sanders, J. K. M. *J. Chem. Soc. Chem. Commun.* **1993**, No. 5, 458–460. doi:10.1039/C39930000458
(84) Breslow, R.; Gabriele, B.; Yang, J. *Tetrahedron Lett.* **1998**, *39*, 2887–2890. doi:10.1016/S0040-4039(98)00425-0
(85) Kaanumalle, L. S.; Gibb, C. L. D.; Gibb, B. C.; Ramamurthy, V. *J. Am. Chem. Soc.* **2004**, *126*, 14366–14367. doi:10.1021/JA0450197
(86) Chao, Y.; Cram, D. J. *J. Am. Chem. Soc.* **1976**, *98*, 1015–1017. doi:10.1021/JA00420A026
(87) Cram, D. J.; Katz, H. E.; Dicker, I. B. *J. Am. Chem. Soc.* **1984**, *106*, 4987–5000. doi:10.1021/JA00329A059

(88) Cram, D. J.; Lam, P. Y. S.; Ho, S. P. *J. Am. Chem. Soc.* **1986**, *108*, 839–841. doi:10.1021/JA00264A048
(89) Breslow, R. *Acc. Chem. Res.* **1995**, *28*, 146–153. doi:10.1021/AR00051A008
(90) Breslow, Ronald. *Artificial Enzymes*; Wiley-VCH, 2006. doi:10.1002/3527606645
(91) Breslow, R. *Science* **1982**, *218*, 532–537. doi:10.1126/science.7123255
(92) Breslow, R.; Czarnik, A. W.; Lauer, M.; Leppkes, R.; Winkler, J.; Zimmerman, S. *J. Am. Chem. Soc.* **1986**, *108*, 1969–1979. doi:10.1021/JA00268A040
(93) Mattei, P.; Diederich, F. *Angew. Chem. Int. Ed.* **1996**, *35*, 1341–1344. doi:10.1002/ANIE.199613411
(94) Lehn, J. M.; Sirlin, C. *J. Chem. Soc. Chem. Commun.* **1978**, No. 21, 949–951. doi:10.1039/C39780000949
(95) Tsao, B. L.; Pieters, R. J.; Rebek, J. *J. Am. Chem. Soc.* **1995**, *117*, 2210–2213. doi:10.1021/JA00113A010
(96) Mackay, L. G.; Wylie, R. S.; Sanders, J. K. M. *J. Am. Chem. Soc.* **1994**, *116*, 3141–3142. doi:10.1021/JA00086A061
(97) Thordarson, P.; Bijsterveld, E. J. A.; Rowan, A. E.; Nolte, R. J. M. *Nature* **2003**, *424*, 915–918. doi:10.1038/Nature01925
(98) Hooley, R. J.; Rebek, J. *Chem. Biol.* **2009**, *16*, 255–264. doi:10.1016/j.chembiol.2008.09.015
(99) Fried, S. D.; Boxer, S. G. *Annu. Rev. Biochem.* **2017**, *86*, 387–415. doi:10.1146/annurev-biochem
(100) Kirby, A. J.; Hollfelder, F. *From Enzyme Models to Model Enzymes*; The Royal Society of Chemistry, 2009. doi:10.1039/9781847559784
(101) Breslow, R.; Zhang, B. *J. Am. Chem. Soc.* **1992**, *114*, 5882–5883. doi:10.1021/JA00040A073
(102) Breslow, R.; Zhang, B. *J. Am. Chem. Soc.* **1994**, *116*, 7893–7894. doi:10.1021/JA00096A055
(103) Shinobu, A.; Agmon, N. *J. Phys. Chem. A* **2009**, *113*, 7253–7266. doi:10.1021/JP8102047
(104) Cui, Q.; Karplus, M. *J. Phys. Chem. B* **2003**, *107*, 1071–1078. doi:10.1021/JP021931V
(105) Li, T. R.; Huck, F.; Piccini, G. M.; Tiefenbacher, K. *Nat. Chem.* **2022**, *14*, 985–994. doi:10.1038/s41557-022-00981-6
(106) Offenbacher, A. R.; Barry, B. A. *J. Phys. Chem. B* **2020**, *124*, 345–354. doi:10.1021/ACS.JPCB.9B08587
(107) Frushicheva, M. P.; Mukherjee, S.; Warshel, A. *J. Phys. Chem. B* **2012**, *116*, 13353–13360. doi:10.1021/JP3084327
(108) Rebek, J. *Angew. Chem. Int. Ed.* **1990**, *29*, 245–255. doi:10.1002/anie.199002451
(109) Nowick, J. S.; Ballester, P.; Ebmeyer, F.; Rebek, J. *J. Am. Chem. Soc.* **1990**, *112*, 8902–8906. doi:10.1021/JA00180A038
(110) Wolfe, J.; Nemeth, D.; Costero, A.; Rebek, J. *J. Am. Chem. Soc.* **1988**, *110*. doi:10.1021/ja00211a057
(111) Cox, C. J. T.; Hale, J.; Molinska, P.; Lewis, J. E. M. *Chem. Soc. Rev.* **2024**. doi:10.1039/D4CS00761A
(112) Wyler, R.; de Mendoza, J.; Rebek, J. *Angew. Chem. Int. Ed.* **1993**, *32*, 1699–1701. doi:10.1002/ANIE.199316991
(113) Kang, J.; Rebek, J. *Nature* **1997**, *385*, 50–52. doi:10.1038/385050a0
(114) Kang, J.; Hilmersson, G.; Santamaría, J.; Rebek, J. *J. Am. Chem. Soc.* **1997**, *20*, 3650–3656. doi:10.1021/JA973898
(115) Rebek, J.; *Angew. Chem. Int. Ed.* **2005**, *44*, 2068–2078. doi:10.1002/ANIE.200462839
(116) Conn, M. M.; Rebek, J. *Chem. Rev.* **1997**, *97*, 1647–1668. doi:10.1021/CR9603800
(117) Zhang, K. Da; Ajami, D.; Rebek, J. *J. Am. Chem. Soc.* **2013**, *135*, 18064–18066. doi:10.1021/ja410644p
(118) Hermann, K.; Ruan, Y.; Hardin, A. M.; Hadad, C. M.; Badjić, J. D. *Chem. Soc. Rev.* **2014**, *44*, 500–514. doi:10.1039/C4CS00140K
(119) Kobayashi, K.; Yamanaka, M. *Chem. Soc. Rev.* **2014**, *44*, 449–466. doi:10.1039/C4CS00153B
(120) Kang, J.; Santamaria, J.; Hilmersson, G.; Rebek J., *J. Am. Chem. Soc.* **1998**, *120*, 7389–7390. doi:10.1021/JA980927N
(121) Chen, J.; Rebek, J. *Org. Lett.* **2002**, *4*, 327–329. doi:10.1021/OL0168115
(122) MacGillivray, L. R.; Atwood, J. L. *Nature* **1997**, *389*, 469–472. doi:10.1038/38985
(123) Borsato, G.; Scarso, A. *Organic Nanoreactors: From Molecular to Supramolecular Organic Compounds*; Academic Press, **2016**, 203–234. doi:10.1016/B978-0-12-801713-5.00007-0

(124) Avram, L.; Cohen, Y.; Rebek, J. *Chem. Commun.* **2011**, *47*, 5368–5375. doi:10.1039/C1CC10150A
(125) Zhang, Q.; Tiefenbacher, K. *J. Am. Chem. Soc.* **2013**, *135*, 16213–16219. doi:10.1021/JA4080375
(126) Zhang, Q.; Tiefenbacher, K. *Nat. Chem.* **2015**, *7*, 197–202. doi:10.1038/nchem.2181
(127) Catti, L.; Zhang, Q.; Tiefenbacher, K. *Chem. Eur. J.* **2016**, *22*, 9060–9066. doi:10.1002/chem.201600726
(128) Zhang, Q.; Catti, L.; Tiefenbacher, K. *Acc. Chem. Res.* **2018**, *51*, 2107–2114. doi:https://doi.org/10.1021/acs.accounts.8b00320
(129) Syntrivanis, L. D.; Némethová, I.; Schmid, D.; Levi, S.; Prescimone, A.; Bissegger, F.; Major, D. T.; Tiefenbacher, K. *J. Am. Chem. Soc.* **2020**, *142*, 5894–5900. doi:10.1021/jacs.0c01464
(130) Merget, S.; Catti, L.; Piccini, G.; Tiefenbacher, K. *J. Am. Chem. Soc.* **2020**, *142*, 4400–4410. doi:10.1021/jacs.9b13239
(131) Heilmann, M.; Knezevic, M.; Piccini, G.; Tiefenbacher, K. *Org. Biomol. Chem.* **2021**, *19*, 3628–3633. doi:10.1039/d1ob00379h
(132) Li, T. R.; Piccini, G. M.; Tiefenbacher, K. *J. Am. Chem. Soc.* **2022**, *145*, 4294–4303. doi:10.1021/JACS.2C13641
(133) Némethová, I.; Schmid, D.; Tiefenbacher, K. *Angew. Chem. Int. Ed.* **2023**, *62*, e202218625. doi:10.1002/ANIE.202218625
(134) Bräuer, T. M.; Zhang, Q.; Tiefenbacher, K. *J. Am. Chem. Soc.* **2017**, *139*, 17500–17507. doi:10.1021/JACS.7B08976
(135) Li, T. R.; Das, C.; Cornu, I.; Prescimone, A.; Piccini, G. M.; Tiefenbacher, K. *JACS Au* **2024**, 4, 1901–1910. doi:10.1021/JACSAU.4C00097
(136) Wang, K.; Cai, X.; Yao, W.; Tang, D.; Kataria, R.; Ashbaugh, H. S.; Byers, L. D.; Gibb, B. C. *J. Am. Chem. Soc.* **2019**, *141*, 6740–6747. doi:10.1021/JACS.9B02287
(137) Yao, W.; Wang, K.; Ismaiel, Y. A.; Wang, R.; Cai, X.; Teeler, M.; Gibb, B. C. *J. Phys. Chem. B* **2021**, *125*, 9333–9340. doi:10.1021/ACS.JPCB.1C05238
(138) Xue, Y.; Hang, X.; Ding, J.; Li, B.; Zhu, R.; Pang, H.; Xu, Q. *Coord. Chem. Rev.* **2021**, *430*, 213656. doi:10.1016/J.CCR.2020.213656
(139) Severinsen, R. J.; Rowlands, G. J.; Plieger, P. G. *J. Incl. Phenom. Macrocycl. Chem.* **2020**, *96*, 29–42. doi:10.1007/s10847-019-00964-0
(140) Piskorz, T. K.; Martí-Centelles, V.; Spicer, R. L.; Duarte, F.; Lusby, P. J. *Chem. Sci.* **2023**, *14*, 11300–11331. doi:10.1039/D3SC02586A
(141) Sinha, I.; Mukherjee, P. S. *Inorg. Chem.* **2018**, *57*, 4205–4221. doi:10.1021/acs.inorgchem.7b03067
(142) Bolliger, J. L.; Belenguer, A. M.; Nitschke, J. R. *Angew. Chem. Int. Ed.* **2013**, *52*, 7958–7962. doi:10.1002/ANIE.201302136
(143) Zhang, D.; Ronson, T. K.; Zou, Y. Q.; Nitschke, J. R. *Nat. Rev. Chem.* **2021**, *5*, 168–182. doi:10.1038/s41570-020-00246-1
(144) Zou, Y. Q.; Zhang, D.; Ronson, T. K.; Tarzia, A.; Lu, Z.; Jelfs, K. E.; Nitschke, J. R. *J. Am. Chem. Soc.* **2021**, *143*, 9009–9015. doi:10.1021/JACS.1C05172
(145) Farrell, J. R.; Mirkin, C. A.; Guzei, I. A.; Liable-Sands, L. M.; Rheingold, A. L. *Angew. Chem. Int. Ed.* **1998**, *37*, 465–467. doi:10.1002/(sici)1521-3773(19980302)37:4<465::aid-anie465>3.0.co;2-a
(146) Martín Díaz, A. E.; Lewis, J. E. M. *Front. Chem.* **2021**, *9*, 706462. doi:10.3389/FCHEM.2021.706462
(147) Dai, F.; He, H.; Xie, A.; Chu, G.; Sun, D.; Ke, Y. *CrystEngComm* **2009**, *11*, 47–49. doi:10.1039/B816015P
(148) Seidel, S. R.; Stang, P. J. *Acc. Chem. Res.* **2002**, *35*, 972–983. doi:10.1021/AR010142D
(149) McConnell, A. J. *Chem. Soc. Rev.* **2022**, *51*, 2957–2971. doi:10.1039/D1CS01143J
(150) Stricklen, P. M.; Volcko, E. J.; Verkade, J. G. *J. Am. Chem. Soc.* **1983**, *105*, 2494–2495. doi:10.1021/JA00346A076
(151) Fujita, M.; Ogura, K. *Bull. Chem. Soc. Jpn.* **1996**, *69*, 1471–1482. doi:10.1246/bcsj.69.1471
(152) Fujita, M.; Yazaki, J.; Ogura, K. *J. Am. Chem. Soc.* **1990**, *112*, 5645–5647. doi:10.1021/JA00170A042
(153) Fujita, M.; Nagao, S.; Ogura, K. *J. Am. Chem. Soc.* **1995**, *117*, 1649–1650. doi:10.1021/ja00110a026

(154) Fujita, M.; Ogura, K. *Coord. Chem. Rev.* **1996**, *148*, 249–264. doi:10.1016/0010-8545(95)01212-5
(155) Beissel, T.; Powers, R. E.; Raymond, K. N. *Angew. Chem. Int. Ed.* **1996**, *35*, 1084-1086. doi:10.1002/anie.199610841
(156) Percástegui, E. G.; Ronson, T. K.; Nitschke, J. R. *Chem. Rev.* **2020**, *120*, 13480–13544. doi:10.1021/acs.chemrev.0c00672
(157) Raee, E.; Yang, Y.; Liu, T. *Giant* **2021**, *5*, 100050. doi:10.1016/J.GIANT.2021.100050
(158) Wiester, M. J.; Ulmann, P. A.; Mirkin, C. A. *Angew. Chem. Int. Ed.* **2011**, *50*, 114–137. doi:10.1002/ANIE.201000380
(159) Samanta, D.; Mukherjee, S.; Patil, Y. P.; Mukherjee, P. S. *Chem. Eur. J.* **2012**, *18*, 12322–12329. doi:10.1002/CHEM.201201679
(160) Yoshizawa, M.; Sato, N.; Fujita, M. *Chem. Lett.* **2005**, *34*, 1392–1393. doi:10.1246/CL.2005.1392
(161) Cullen, W.; Metherell, A. J.; Wragg, A. B.; Taylor, C. G. P.; Williams, N. H.; Ward, M. D. *J. Am. Chem. Soc.* **2018**, 140, 2821–2828. doi:10.1021/JACS.7B11334
(162) Taylor, C. G. P.; Metherell, A. J.; Argent, S. P.; Ashour, F. M.; Williams, N. H.; Ward, M. D. *Chem. Eur. J.* **2020**, 26, 3065–3073. doi:10.1002/CHEM.201904708
(163) Cullen, W.; Misuraca, M. C.; Hunter, C. A.; Williams, N. H.; Ward, M. D. *Nat. Chem.* **2016**, *8*, 231–236. doi:10.1038/nchem.2452
(164) Ngai, C.; Wu, H. T.; da Camara, B.; Williams, C. G.; Mueller, L. J.; Julian, R. R.; Hooley, R. J. *Angew. Chem. Int. Ed.* **2022**, *61*, e202117011. doi:10.1002/ANIE.202117011
(165) Leung, D. H.; Bergman, R. G.; Raymond, K. N. *J. Am. Chem. Soc.* **2007**, *129*, 2746–2747. doi:10.1021/JA068688O
(166) Martí-Centelles, V.; Lawrence, A. L.; Lusby, P. J. *J. Am. Chem. Soc.* **2018**, *140*, 2862–2868. doi:10.1021/jacs.7b12146
(167) Ngai, C.; Da Camara, B.; Woods, C. Z.; Hooley, R. J. *J. Org. Chem.* **2021**, *86*, 12862–12871. doi:10.1021/acs.joc.1c01511
(168) Kaphan, D. M.; Toste, F. D.; Bergman, R. G.; Raymond, K. N. *J. Am. Chem. Soc.* **2015**, *137*, 9202–9205. doi:10.1021/JACS.5B01261
(169) Bierschenk, S. M.; Bergman, R. G.; Raymond, K. N.; Toste, F. D. *J. Am. Chem. Soc.* **2020**, *142*, 733–737. doi:10.1021/JACS.9B13177
(170) Takezawa, H.; Shitozawa, K.; Fujita, M. *Nat. Chem.* **2020**, *12*, 574–578. doi:10.1038/s41557-020-0455-y
(171) Wang, H.; Zhang, Y.; Ji, G.; Wei, J.; Zhao, L.; He, C.; Duan, C. *J. Am. Chem. Soc.* **2024**, *146*, 29272–29277. doi:10.1021/JACS.4C08547
(172) McGrath, D. V; Grubbs, R. H.; Ziller, J. W.; Walter, C. J.; Anderson, H. L.; M Sanders, J. K. *Chem. Lett.* **2000**, *29*, 598–599. doi:10.1246/CL.2000.598
(173) Xu, M.; Sun, B.; Poole, D. A.; Bobylev, E. O.; Jing, X.; Wu, J.; He, C.; Duan, C.; Reek, J. N. H. *Chem. Sci.* **2023**, *14*, 11699–11707. doi:10.1039/D3SC02998K
(174) Merlau, M. L.; Mejia, M. D. P.; Nguyen, S. T.; Hupp, J. T. *Angew. Chem. Int. Ed.* **2001**, *40*, 4239-4242. doi:10.1002/1521-3773(20011119)40:22<4239::AID-ANIE4239>3.0.CO;2-E
(175) Howlader, P.; Das, P.; Zangrando, E.; Mukherjee, P. S. *J. Am. Chem. Soc.* **2016**, *138*, 1668–1676. doi:10.1021/JACS.5B12237
(176) Hastings, C. J.; Pluth, M. D.; Bergman, R. G.; Raymond, K. N. *J. Am. Chem. Soc.* **2010**, *132*, 6938–6940. doi:10.1021/ja102633e
(177) Wang, Z. J.; Brown, C. J.; Bergman, R. G.; Raymond, K. N.; Toste, F. D. *J. Am. Chem. Soc.* **2011**, *133*, 7358–7360. doi:10.1021/JA202055V
(178) Pluth, M. D.; Bergman, R. G.; Raymond, K. N. *J. Org. Chem.* **2009**, *74*, 58–63. doi:10.1021/JO802131V
(179) Davis, A. V.; Fiedler, D.; Ziegler, M.; Terpin, A.; Raymond, K. N. *J. Am. Chem. Soc.* **2007**, *129*, 15354–15363. doi:10.1021/ja0764815
(180) Di Stefano, S.; Cacciapaglia, R.; Mandolini, L. *Eur. J. Org. Chem.* **2014**, *2014*, 7304–7315. doi:10.1002/EJOC.201402690
(181) Hong, C. M.; Morimoto, M.; Kapustin, E. A.; Alzakhem, N.; Bergman, R. G.; Raymond, K. N.; Toste, F. D. *J. Am. Chem. Soc.* **2018**, *140*, 6591–6595. doi:10.1021/JACS.8B01701
(182) Ohara, K.; Kawano, M.; Inokuma, Y.; Fujita, M. *J. Am. Chem. Soc.* **2010**, *132*, 30–31. doi:10.1021/JA908794N

(183) Sunohara, H.; Koyamada, K.; Takezawa, H.; Fujita, M. *Chem. Commun.* **2021**, *57*, 9300–9302. doi:10.1039/D1CC03620C
(184) Murase, T.; Nishijima, Y.; Fujita, M. *J. Am. Chem. Soc.* **2012**, *134*, 162–164. doi:10.1021/JA210068F
(185) Yoshizawa, M.; Miyagi, S.; Kawano, M.; Ishiguro, K.; Fujita, M. *J. Am. Chem. Soc.* **2004**, *126*, 9172–9173. doi:10.1021/JA047612U
(186) Yoshizawa, M.; Tamura, M.; Fujita, M. *Science* **2006**, *312*, 251–254. doi:10.1126/science.1124985
(187) Takezawa, H.; Kanda, T.; Nanjo, H.; Fujita, M. *J. Am. Chem. Soc.* **2019**, *141*, 5112–5115. doi:10.1021/JACS.9B00131
(188) Takezawa, H.; Iizuka, K.; Fujita, M. *Angew. Chem. Int. Ed.* **2024**, *63*, e202319140. doi:10.1002/ANIE.202319140
(189) Ueda, Y.; Ito, H.; Fujita, D.; Fujita, M. *J. Am. Chem. Soc.* **2017**, *139*, 6090–6093. doi:10.1021/jacs.7b02745
(190) Martí-Centelles, V.; Duarte, F.; Lusby, P. J. *Isr. J. Chem.* **2019**, *59*, 257–266. doi:10.1002/IJCH.201800106
(191) Martí-Centelles, V.; Spicer, R. L.; Lusby, P. J. *Chem. Sci.* **2020**, *11*, 3236–3240. doi:10.1039/D0SC00341G
(192) Wang, J.; Young, T. A.; Duarte, F.; Lusby, P. J. *J. Am. Chem. Soc.* **2020**, *142*, 17743–17750. doi:10.1021/jacs.0c08639
(193) Young, T. A.; Martí-Centelles, V.; Wang, J.; Lusby, P. J.; Duarte, F. *J. Am. Chem. Soc.* **2020**, *142*, 1300–1310. doi:10.1021/jacs.9b10302
(194) Spicer, R. L.; Stergiou, A. D.; Young, T. A.; Duarte, F.; Symes, M. D.; Lusby, P. J. *J. Am. Chem. Soc.* **2020**, *142*, 2134–2139. doi:10.1021/jacs.9b11273
(195) Boaler, P. J.; Piskorz, T. K.; Bickerton, L. E.; Wang, J.; Duarte, F.; Lloyd-Jones, G. C.; Lusby, P. J. *J. Am. Chem. Soc.* **2024**, *146*, 19317–19326. doi:10.1021/JACS.4C05160
(196) DiNardi, R. G.; Rasheed, S.; Capomolla, S. S.; Chak, M. H.; Middleton, I. A.; Macreadie, L. K.; Violi, J. P.; Donald, W. A.; Lusby, P. J.; Beves, J. E. *J. Am. Chem. Soc.* **2024**, *146*, 21196–21202. doi:10.1021/JACS.4C04846
(197) Holloway, L. R.; Bogie, P. M.; Lyon, Y.; Ngai, C.; Miller, T. F.; Julian, R. R.; Hooley, R. J. *J. Am. Chem. Soc.* **2018**, *140*, 8078–8081. doi:10.1021/jacs.8b03984
(198) Wang, Q. Q.; Gonell, S.; Leenders, S. H. A. M.; Dürr, M.; Ivanovic-Burmazovic, I.; Reek, J. N. H. *Nat. Chem.* **2016**, *8*, 225–230. doi:10.1038/nchem.2425
(199) Wang, Z. J.; Clary, K. N.; Bergman, R. G.; Raymond, K. N.; Toste, F. D. *Nat. Chem.* **2013**, *5*, 100–103. doi:10.1038/nchem.1531
(200) Zarra, S.; Wood, D. M.; Roberts, D. A.; Nitschke, J. R. *Chem. Soc. Rev.* **2014**, *44*, 419–432. doi:10.1039/C4CS00165F
(201) Salles, A. G.; Zarra, S.; Turner, R. M.; Nitschke, J. R. *J. Am. Chem. Soc.* **2013**, *135*, 19143–19146. doi:10.1021/ja412235e
(202) Roberts, D. A.; Pilgrim, B. S.; Nitschke, J. R. *Chem. Soc. Rev.* **2018**, *47*, 626–644. doi:10.1039/C6CS00907G
(203) McTernan, C. T.; Ronson, T. K.; Nitschke, J. R. *J. Am. Chem. Soc.* **2019**, *141*, 6837–6842. doi:10.1021/jacs.9b02604
(204) Black, M. R.; Bhattacharyya, S.; Argent, S. P.; Pilgrim, B. S. *J. Am. Chem. Soc.* **2024**, *146*, 28233–28241. doi:10.1021/JACS.4C08591
(205) Wu, K.; Benchimol, E.; Baksi, A.; Clever, G. H. *Nat. Chem.* **2024**, *16*, 584–591. doi:10.1038/s41557-023-01415-7
(206) Meng, W.; Ronson, T. K.; Nitschke, J. R. *Proc. Natl. Acad. Sci.* **2013**, *110*, 10531–10535. doi:10.1073/pnas.1302683110
(207 Pullen, S.; Tessarolo, J.; Clever, G. H. *Chem. Sci.* **2021**, *12*, 7269–7293. doi:10.1039/D1SC01226F
(208) Tarzia, A.; Lewis, J. E. M.; Jelfs, K. E. *Angew. Chem. Int. Ed.* **2021**, *60*, 20879–20887. doi:10.1002/anie.202106721
(209) Lewis, J. E. M. *Angew. Chem. Int. Ed.* **2022**, *61*, e202212392. doi:10.1002/ANIE.202212392
(210) Lewis, J. E. M.; Crowley, J. D. *Chempluschem* **2020**, *85*, 815–827. doi:10.1002/CPLU.202000153

(211) Faulkner, L. A. V.; Crowley, J. D. *Chem* **2024**, *10*, 440–442. doi:10.1016/J.CHEMPR.2024.01.015
(212) Pearcy, A. C.; Lisboa, L. S.; Preston, D.; Page, N. B.; Lawrence, T.; Wright, L. J.; Hartinger, C. G.; Crowley, J. D. *Chem. Sci.* **2023**, *14*, 8615–8623. doi:10.1039/D3SC01354E
(213) Vasdev, R. A. S.; Preston, D.; Casey-Stevens, C. A.; Martí-Centelles, V.; Lusby, P. J.; Garden, A. L.; Crowley, J. D. *Inorg. Chem.* **2023**, *62*, 1833–1844. doi:10.1021/ACS.INORGCHEM.2C00937
(214) Li, R. J.; Tarzia, A.; Posligua, V.; Jelfs, K. E.; Sanchez, N.; Marcus, A.; Baksi, A.; Clever, G. H.; Fadaei-Tirani, F.; Severin, K. *Chem. Sci.* **2022**, *13*, 11912–11917. doi:10.1039/D2SC03856K
(215) McTernan, C. T.; Davies, J. A.; Nitschke, J. R. *Chem. Rev.* **2022**, *122*, 10393–10437. doi:10.1021/ACS.CHEMREV.1C00763/
(216) Li, R. J.; Holstein, J. J.; Hiller, W. G.; Andréasson, J.; Clever, G. H. *J. Am. Chem. Soc.* **2019**, *141*, 2097–2103. doi:10.1021/JACS.8B11872
(217) Barber, B. E.; Jamieson, E. M. G.; White, L. E. M.; McTernan, C. T. *Chem* **2024**, *10*, 2792-2806. doi:10.1016/J.CHEMPR.2024.05.002
(218) Hoskins, B. F.; Robson, R. *J. Am. Chem. Soc.* **1990**, *112*, 1546–1554. doi:10.1021/JA00160A038
(219) Yaghi, O. M.; Li, G.; Li, H. *Nature* **1995**, *378*, 703–706. doi:10.1038/378703a0
(220) Liu, G.; Sheng, J.; Zhao, Y. *Sci. China Chem.* **2017**, *60*, 1015–1022. doi:10.1007/s11426-017-9070-1
(221) Li, H.; Pan, Q.; Ma, Y.; Guan, X.; Xue, M.; Fang, Q.; Yan, Y.; Valtchev, V.; Qiu, S. *J. Am. Chem. Soc.* **2016**, *138*, 14783–14788. doi:10.1021/jacs.6b09563
(222) Hu, H.; Yan, Q.; Ge, R.; Gao, Y. *Chin. J. Catal.* **2018**, *39*, 1167–1179. doi:10.1016/S1872-2067(18)63057-8
(223) Côté, A. P.; Benin, A. I.; Ockwig, N. W.; O'Keeffe, M.; Matzger, A. J.; Yaghi, O. M. *Science* **2005**, *310*, 1166–1170. doi:10.1126/SCIENCE.1120411
(224) Diercks, C. S.; Yaghi, O. M. *Science* **2017**, *355*, 923–931. doi:10.1126/science.aal1585
(225) Hu, A.; Ngo, H. L.; Lin, W. *Angew. Chem. Int. Ed.* **2003**, *115*, 6182–6185. doi:10.1002/ANGE.200351264
(226) Guo, J.; Jiang, D. *ACS Cent. Sci.* **2020**, *6*, 869–879. doi:10.1021/ACSCENTSCI.0C00463
(227) Ma, L.; Abney, C.; Lin, W. *Chem. Soc. Rev.* **2009**, *38*, 1248–1256. doi:10.1039/B807083K
(228) Rogge, S. M. J.; Bavykina, A.; Hajek, J.; Garcia, H.; Olivos-Suarez, A. I.; Sepúlveda-Escribano, A.; Vimont, A.; Clet, G.; Bazin, P.; Kapteijn, F.; Daturi, M.; Ramos-Fernandez, E. V.; Llabrés Xamena, F. X. I.; Van Speybroeck, V.; Gascon, J. *Chem. Soc. Rev.* **2017**, *46*, 3134–3184. doi:10.1039/c7cs00033b
(229) Wu, Y.; Xu, H.; Chen, X.; Gao, J.; Jiang, D. *Chem. Commun.* **2015**, *51*, 10096–10098. doi:10.1039/C5CC03457D
(230) Wang, K. Y.; Zhang, J.; Hsu, Y. C.; Lin, H.; Han, Z.; Pang, J.; Yang, Z.; Liang, R. R.; Shi, W.; Zhou, H. C. *Chem. Rev.* **2023**, *123*, 5347–5420. doi:10.1021/acs.chemrev.2c00879
(231) Seo, J. S.; Whang, D.; Lee, H.; Jun, S. I.; Oh, J.; Jeon, Y. J.; Kim, K. *Nature* **2000**, *404*, 982–986. doi:10.1038/35010088
(232) Ma, M.; Chen, J.; Liu, H.; Huang, Z.; Huang, F.; Li, Q.; Xu, Y. *Nanoscale* **2022**, *14*, 13405–13427. doi:10.1039/d2nr01772e
(233) Xu, H.; Gao, J.; Jiang, D. *Nat. Chem.* **2015**, *7*, 905–912. doi:10.1038/nchem.2352
(234) Dhakshinamoorthy, A.; Asiri, A. M.; Garcia, H. *Chem. Eur. J.* **2023**, *29*, e202204016. doi:10.1002/CHEM.202204016
(235) Montà-González, G.; Sancenón, F.; Martínez-Máñez, R.; Martí-Centelles, V. *Chem. Rev.* **2022**, *122*, 13636–13708. doi:10.1021/acs.chemrev.2c00198
(236) Jia, F.; He, Z.; Yang, L. P.; Pan, Z. S.; Yi, M.; Jiang, R. W.; Jiang, W. *Chem. Sci.* **2015**, *6*, 6731–6738. doi:10.1039/C5SC03251B
(237) Dietrich, B.; Lehn, J. M.; Sauvage, J. P. *Tet. Lett.* **1969**, *10*, 2885–2888. doi:10.1016/S0040-4039(01)88299-X
(238) Zhang, C.; Chen, C. F. *J. Org. Chem.* **2007**, *72*, 9339–9341. doi:10.1021/JO7017526
(239) Avellaneda, A.; Valente, P.; Burgun, A.; Evans, J. D.; Markwell-Heys, A. W.; Rankine, D.; Nielsen, D. J.; Hill, M. R.; Sumby, C. J.; Doonan, C. J. *Angew. Chem., Int. Ed.* **2013**, *52*, 3746–3749. doi:10.1002/ANIE.201209922

(240) Burgun, A.; Valente, P.; Evans, J. D.; Huang, D. M.; Sumby, C. J.; Doonan, C. J. *Chem. Comm.* **2016**, *52*, 8850–8853. doi:10.1039/C6CC04423A
(241) Kajiyama, K.; Tsurumaki, E.; Wakamatsu, K.; Fukuhara, G.; Toyota, S. *Chempluschem* **2021**, *86*, 716–722. doi:10.1002/CPLU.202000816
(242) Kayahara, E.; Iwamoto, T.; Takaya, H.; Suzuki, T.; Fujitsuka, M.; Majima, T.; Yasuda, N.; Matsuyama, N.; Seki, S.; Yamago, S. *Nat. Comm.* **2013**, *4*, 2694. doi:10.1038/ncomms3694
(243) Matsui, K.; Segawa, Y.; Namikawa, T.; Kamada, K.; Itami, K. *Chem. Sci.* **2012**, *4*, 84–88. doi:10.1039/C2SC21322B
(244) Matsui, K.; Segawa, Y.; Itami, K. *J. Am. Chem. Soc.* **2014**, *136*, 16452–16458. doi:10.1021/JA509880V
(245) Zhao, X.; Liu, Y.; Zhang, Z. Y.; Wang, Y.; Jia, X.; Li, C. *Angew. Chem., Int. Ed.* **2021**, *60*, 17904–17909. doi:10.1002/ANIE.202104875
(246) Ni, Y.; Gopalakrishna, T. Y.; Phan, H.; Kim, T.; Herng, T. S.; Han, Y.; Tao, T.; Ding, J.; Kim, D.; Wu, J. *Nat. Chem.* **2020**, *12*, 242–248. doi:10.1038/s41557-019-0399-2
(247) Gu, X.; Gopalakrishna, T. Y.; Phan, H.; Ni, Y.; Herng, T. S.; Ding, J.; Wu, J. *Angew. Chem., Int. Ed.* **2017**, *56*, 15383–15387. doi:10.1002/ANIE.201709537
(248) Liu, Y.; Zhao, W.; Chen, C. H.; Flood, A. H. *Science* **2019**, *365*, 159–161. doi:10.1126/SCIENCE.AAW5145
(249) Galán, A.; Escudero-Adán, E. C.; Ballester, P. *Chem. Sci.* **2017**, *8*, 7746–7750. doi:10.1039/C7SC03731G
(250) Zhang, L.; Das, R.; Li, C. T.; Wang, Y. Y.; Hahn, F. E.; Hua, K.; Sun, L. Y.; Han, Y. F. *Angew. Chem., Int. Ed.* **2019**, *58*, 13360–13364. doi:10.1002/ANIE.201907003
(251) Rowan, S. J.; Cantrill, S. J.; Cousins, G. R. L.; Sanders, J. K. M.; Stoddart, J. F. *Angew. Chem., Int. Ed.* **2002**, *41*, 898–952. doi:10.1002/1521-3773(20020315)41:6<898::AID-ANIE898>3.0.CO;2-E
(252) Jin, Y.; Yu, C.; Denman, R. J.; Zhang, W. *Chem. Soc. Rev.* **2013**, *42*, 6634–6654. doi:10.1039/C3CS60044K
(253) Hamilton, D. G.; Feeder, N.; Teat, S. J.; Sanders, J. K. M. *New J. Chem.* **1998**, *22*, 1019–1021. doi:10.1039/A805505J
(254) Lehn, J. M. *Chem. Soc. Rev.* **2007**, *36*, 151–160. doi:10.1039/B616752G
(255) Cougnon, F. B. L.; Sanders, J. K. M. *Acc. Chem. Res.* **2012**, *45*, 2211–2221. doi:10.1021/ar200240m
(256) Ono, K.; Iwasawa, N. *Chem. Eur. J.* **2018**, *24*, 17856–17868. doi:10.1002/CHEM.201802253
(257) Beuerle, F.; Klotzbach, S.; Dhara, A. *Synlett* **2016**, *27*, 1133–1138. doi:10.1055/S-0035-1561364
(258) Jin, Y.; Wang, Q.; Taynton, P.; Zhang, W. *Acc. Chem. Res.* **2014**, *47*, 1575–1586. doi:10.1021/AR500037V
(259) Gayen, K. S.; Das, T.; Chatterjee, N. *Eur. J. Org. Chem.* **2021**, *2021*, 861–876. doi:10.1002/EJOC.202001194
(260) Acharyya, K.; Mukherjee, P. S. *Angew. Chem. Int. Ed.* **2019**, *58*, 8640–8653. doi:10.1002/anie.201900163
(261) Acharyya, K.; Mukherjee, S.; Mukherjee, P. S. *J. Am. Chem. Soc.* **2013**, *135*, 554–557. doi:10.1021/ja310083p
(262) Bhandari, P.; Mukherjee, P. S. *Chem. Eur. J.* **2022**, *28*, e202201901. doi:10.1002/CHEM.202201901
(263) Acharyya, K.; Mukherjee, P. S. *Chem. Commun.* **2014**, *50*, 15788–15791. doi:10.1039/C4CC06225F
(264) Acharyya, K.; Mukherjee, P. S. *Chem. Commun.* **2015**, *51*, 4241–4244
(265) Ro, S.; Rowan, S. J.; Pease, A. R.; Cram, D. J.; Stoddart, J. F. *Org. Lett.* **2000**, *2*, 2411–2414. doi:10.1021/OL005962P
(266) Hong, S.; Rohman, M. R.; Jia, J.; Kim, Y.; Moon, D.; Kim, Y.; Ko, Y. H.; Lee, E.; Kim, K. *Angew. Chem. Int. Ed.* **2015**, *54*, 13241–13244. doi:10.1002/anie.201505531
(267) Wang, H.; Fang, S.; Wu, G.; Lei, Y.; Chen, Q.; Wang, H.; Wu, Y.; Lin, C.; Hong, X.; Kim, S. K.; Sessler, J. L.; Li, H. *J. Am. Chem. Soc.* **2020**, *142*, 20182–20190. doi:10.1021/jacs.0c10253
(268) Liu, Y.; Liu, X.; Warmuth, R. *Chem. Eur. J.* **2007**, *13*, 8953–8959. doi:10.1002/chem.200701067
(269) Bucher, C.; Zimmerman, R. S.; Lynch, V.; Sessler, J. L. *J. Am. Chem. Soc.* **2001**, *123*, 9716–9717. doi:10.1021/ja016629z

(270) Tönnemann, J.; Scopelliti, R.; Zhurov, K. O.; Menin, L.; Dehnen, S.; Severin, K. *Chem. Eur. J.* **2012**, *18*, 9939-9945. doi:10.1002/chem.201201287
(271) Jiao, T.; Chen, L.; Yang, D.; Li, X.; Wu, G.; Zeng, P.; Zhou, A.; Yin, Q.; Pan, Y.; Wu, B.; Hong, X.; Kong, X.; Lynch, V. M.; Sessler, J. L.; Li, H. *Angew. Chem. Int. Ed.* **2017**, *56*, 14545–14550. doi:10.1002/ANIE.201708246
(272) Kudo, H.; Hayashi, R.; Mitani, K.; Yokozawa, T.; Kasuga, N. C.; Nishikubo, T. *Angew. Chem. Int. Ed.* **2006**, *45*, 7948–7952. doi:10.1002/ANIE.200603013
(273) Kudo, H.; Shimoyama, D.; Sekiya, R.; Haino, T. *Chem. Lett.* **2021**, *50*, 825–831. doi:10.1246/CL.200773
(274) Lee, S.; Yang, A.; Moneypenny, T. P.; Moore, J. S. *J. Am. Chem. Soc.* **2016**, *138*, 2182–2185. doi:10.1021/JACS.6B00468
(275) Huang, S.; Lei, Z.; Jin, Y.; Zhang, W. *Chem. Sci.* **2021**, *12*, 9591–9606. doi:10.1039/D1SC01881G
(276) Li, A.; Xiong, S.; Zhou, W.; Zhai, H.; Liu, Y.; He, Q. *Chem. Comm.* **2021**, *57*, 4496–4499. doi:10.1039/D1CC01158H
(277) Bera, S.; Basu, A.; Tothadi, S.; Garai, B.; Banerjee, S.; Vanka, K.; Banerjee, R. *Angew. Chem. Int. Ed.* **2017**, *56*, 2123–2126. doi:10.1002/ANIE.201611260
(278) Long, A.; Lefevre, S.; Guy, L.; Robert, V.; Dutasta, J. P.; Chevallier, M. L.; Della-Negra, O.; Saaidi, P. L.; Martinez, A. *New J. Chem.* **2019**, *43*, 10222–10226. doi:10.1039/C9NJ01674K
(279) Benke, B. P.; Aich, P.; Kim, Y.; Kim, K. L.; Rohman, M. R.; Hong, S.; Hwang, I. C.; Lee, E. H.; Roh, J. H.; Kim, K. *J. Am. Chem. Soc.* **2017**, *139*, 7432–7435. doi:10.1021/JACS.7B02708
(280) Su, K.; Wang, W.; Du, S.; Ji, C.; Yuan, D. *Nat. Comm.* **2021**, *12*, 3703. doi:10.1038/s41467-021-24042-7
(281) Huang, H. H.; Song, K. S.; Prescimone, A.; Aster, A.; Cohen, G.; Mannancherry, R.; Vauthey, E.; Coskun, A.; Šolomek, T. *Chem. Sci.* **2021**, *12*, 5275–5285. doi:10.1039/D1SC00347J
(282) Jędrzejewska, H.; Wielgus, E.; Kaźmierski, S.; Rogala, H.; Wierzbicki, M.; Wróblewska, A.; Pawlak, T.; Potrzebowski, M. J.; Szumna, A. *Chem. Eur. J.* **2020**, *26*, 1558–1566. doi:10.1002/CHEM.201904024
(283) Yuan, Y. Di; Dong, J.; Liu, J.; Zhao, D.; Wu, H.; Zhou, W.; Gan, H. X.; Tong, Y. W.; Jiang, J.; Zhao, D. *Nat. Comm.* **2020**, *11*, 4927. doi:10.1038/s41467-020-18639-7
(284) Koo, J.; Kim, I.; Kim, Y.; Cho, D.; Hwang, I. C.; Mukhopadhyay, R. D.; Song, H.; Ko, Y. H.; Dhamija, A.; Lee, H.; Hwang, W.; Kim, S.; Baik, M. H.; Kim, K. *Chem* **2020**, *6*, 3374–3384. doi:10.1016/J.CHEMPR.2020.10.002
(285) Mastalerz, M. *Chem. Commun.* **2008**, No. 39, 4756. doi:10.1039/b808990f
(286) Mastalerz, M. *Acc. Chem. Res.* **2018**, *51*, 2411–2422. doi:10.1021/acs.accounts.8b00298
(287) Tozawa, T.; Jones, J. T. A.; Swamy, S. I.; Jiang, S.; Adams, D. J.; Shakespeare, S.; Clowes, R.; Bradshaw, D.; Hasell, T.; Chong, S. Y.; Tang, C.; Thompson, S.; Parker, J.; Trewin, A.; Bacsa, J.; Slawin, A. M. Z.; Steiner, A.; Cooper, A. I. *Nat. Mater.* **2009**, *8*, 973–978. doi:10.1038/nmat2545
(288) Little, M. A.; Cooper, A. I. *Adv. Funct. Mater.* **2020**, *30*, 1909842. doi:10.1002/adfm.201909842
(289) Skowronek, P.; Gawronski, J. *Org. Lett.* **2008**, *10*, 4755–4758. doi:10.1021/OL801702J
(290) Whitesides, G. M.; Grzybowski, B. *Science* **2002**, *295*, 2418–2421. doi:10.1126/SCIENCE.1070821/
(291) Stupp, S. I.; Palmer, L. C. *Chem. Mater.* **2014**, *26*, 507–518. doi:10.1021/cm403028b
(292) Lindsey, J. S. *New J. Chem.* **1991**, *15*, 153–180. doi:10.1002/CHIN.199138328
(293) Skowronek, P.; Warzajtis, B.; Rychlewska, U.; Gawroński, J. *Chem. Commun.* **2013**, *49*, 2524–2526. doi:10.1039/C3CC39098E
(294) Samantray, S.; Krishnaswamy, S.; Chand, D. K. *Nat. Commun.* **2020**, *11*, 880. doi:10.1038/s41467-020-14703-4
(295) Cook, T. R.; Stang, P. J. *Chem. Rev.* **2015**, *115*, 7001–7045. doi:10.1021/cr5005666
(296) Chen, Y.; Cao, Z.; Feng, T.; Zhang, X.; Li, Z.; Dong, X.; Huang, S.; Liu, Y.; Cao, X.; Sue, A. C. H.; Peng, C.; Lin, X.; Wang, L.; Li, H. *Angew. Chem. Int. Ed.* **2024**, *63*, e202400467. doi:10.1002/anie.202400467
(297) Holsten, M.; Feierabend, S.; Elbert, S. M.; Rominger, F.; Oeser, T.; Mastalerz, M. *Chem. Eur. J.* **2021**, *27*, 9383–9390. doi:10.1002/chem.202100666
(298) Hasell, T.; Cooper, A. I. *Nat. Rev. Mater.* **2016**, *1*, 16053. doi:10.1038/natrevmats.2016.53
(299) Mastalerz, M. *Chem. Eur. J.* **2012**, *18*, 10082–10091. doi:10.1002/CHEM.201201351
(300) Mastalerz, M. *Synlett* **2013**, *24*, 781–786. doi:10.1055/s-0032-1318328

(301) Schneider, M. W.; Oppel, I. M.; Mastalerz, M. *Chem. Eur. J.* **2012**, *18*, 4156–4160. doi:10.1002/chem.201200032
(302) Brutschy, M.; Schneider, M. W.; Mastalerz, M.; Waldvogel, S. R. *Adv. Mater.* **2012**, *24*, 6049–6052. doi:10.1002/adma.201202786
(303) Brutschy, M.; Schneider, M. W.; Mastalerz, M.; Waldvogel, S. R. *Chem. Commun.* **2013**, *49*, 8398–8400. doi:10.1039/c3cc43829e
(304) Schneider, M. W.; Oppel, I. M.; Griffin, A.; Mastalerz, M. *Angew. Chem. Int. Ed.* **2013**, *52*, 3611–3615. doi:10.1002/anie.201208156
(305) Zhang, G.; Presly, O.; White, F.; Oppel, I. M.; Mastalerz, M. *Angew. Chem. Int. Ed.* **2014**, *53*, 1516–1520. doi:10.1002/anie.201308924
(306) Alexandre, P. E.; Zhang, W.-S.; Rominger, F.; Elbert, S. M.; Schröder, R. R.; Mastalerz, M. *Angew. Chem. Int. Ed.* **2020**, *59*, 19675–19679. doi:10.1002/anie.202007048
(307) Tian, K.; Elbert, S. M.; Hu, X. Y.; Kirschbaum, T.; Zhang, W.-S.; Rominger, F.; Schröder, R. R.; Mastalerz, M. *Adv. Mater.* **2022**, *34*, 2202290. doi:10.1002/adma.202202290
(308) Reiss, P. S.; Little, M. A.; Santolini, V.; Chong, S. Y.; Hasell, T.; Jelfs, K. E.; Briggs, M. E.; Cooper, A. I. *Chem. Eur. J.* **2016**, *22*, 16547–16553. doi:10.1002/chem.201603593
(309) Slater, A. G.; Little, M. A.; Briggs, M. E.; Jelfs, K. E.; Cooper, A. I. *Mol. Syst. Des. Eng.* **2018**, *3*, 223–227. doi:10.1039/c7me00090a
(310) Jiang, S.; Du, Y.; Marcello, M.; Corcoran, E. W.; Calabro, D. C.; Chong, S. Y.; Chen, L.; Clowes, R.; Hasell, T.; Cooper, A. I. *Angew. Chem. Int. Ed.* **2018**, *57*, 11228–11232. doi:10.1002/anie.201803244
(311) Liu, M.; Zhang, L.; Little, M. A.; Kapil, V.; Ceriotti, M.; Yang, S.; Ding, L.; Holden, D. L.; Balderas-Xicohténcatl, R.; He, D.; Clowes, R.; Chong, S. Y.; Schütz, G.; Chen, L.; Hirscher, M.; Cooper, A. I. *Science* **2019**, *366*, 613–620. doi:10.1126/science.aax7427
(312) Jones, J. T. A.; Hasell, T.; Wu, X.; Bacsa, J.; Jelfs, K. E.; Schmidtmann, M.; Chong, S. Y.; Adams, D. J.; Trewin, A.; Schiffman, F.; Cora, F.; Slater, B.; Steiner, A.; Day, G. M.; Cooper, A. I. *Nature* **2011**, *474*, 367–371. doi:10.1038/Nature10125
(313) Hasell, T.; Little, M. A.; Chong, S. Y.; Schmidtmann, M.; Briggs, M. E.; Santolini, V.; Jelfs, K. E.; Cooper, A. I. *Nanoscale* **2017**, *9*, 6783–6790. doi:10.1039/c7nr01301a
(314) Briggs, M. E.; Cooper, A. I. *Chem. Mater.* **2017**, *29*, 149–157. doi:10.1021/acs.chemmater.6b02903
(315) Bhandari, P.; Mukherjee, P. S. *ACS Catal.* **2023**, *13*, 6126–6143. doi:10.1021/acscatal.3c01080
(316) Xu, N.; Su, K.; El-Sayed, E. S. M.; Ju, Z.; Yuan, D. *Chem. Sci.* **2022**, *13*, 3582–3588. doi:10.1039/d2sc00395c
(317) Xu, Y. Y.; Liu, H. K.; Wang, Z. K.; Song, B.; Zhang, D. W.; Wang, H.; Li, Z.; Li, X.; Li, Z. T. *J. Org. Chem.* **2021**, *86*, 3943–3951. doi:10.1021/acs.joc.0c02792
(318) Mondal, B.; Mukherjee, P. S. *J. Am. Chem. Soc.* **2018**, *140*, 12592–12601. doi:10.1021/JACS.8B07767
(319) Zhang, Y.; Xiong, Y.; Ge, J.; Lin, R.; Chen, C.; Peng, Q.; Wang, D.; Li, Y. *Chem. Commun.* **2018**, *54*, 2796–2799. doi:10.1039/C7CC09918E
(320) Sun, J. K.; Zhan, W. W.; Akita, T.; Xu, Q. *J. Am. Chem. Soc.* **2015**, *137*, 7063–7066. doi:10.1021/jacs.5b04029
(321) Yang, X.; Sun, J. K.; Kitta, M.; Pang, H.; Xu, Q. *Nat. Catal.* **2018**, *1*, 214–220. doi:10.1038/s41929-018-0030-8
(322) Jiang, S.; Cox, H. J.; Papaioannou, E. I.; Tang, C.; Liu, H.; Murdoch, B. J.; Gibson, E. K.; Metcalfe, I. S.; Evans, J. S. O.; Beaumont, S. K. *Nanoscale* **2019**, *11*, 14929–14936. doi:10.1039/c9nr04553h
(323) Mondal, B.; Acharyya, K.; Howlader, P.; Mukherjee, P. S. *J. Am. Chem. Soc.* **2016**, *138*. doi:10.1021/jacs.5b13307
(324) Qiu, L.; McCaffrey, R.; Jin, Y.; Gong, Y.; Hu, Y.; Sun, H.; Park, W.; Zhang, W. *Chem. Sci.* **2018**, *9*, 676–680. doi:10.1039/C7SC03148C
(325 Verma, A.; Tomar, K.; Bharadwaj, P. K. *Inorg. Chem.* **2019**, *58*. doi:10.1021/acs.inorgchem.8b03015
(326) Sun, N.; Wang, C.; Wang, H.; Yang, L.; Jin, P.; Zhang, W.; Jiang, J. *Angew. Chem. Int. Ed.* **2019**, *58*, 18011–18016. doi:10.1002/anie.201908703
(327) Perraud, O.; Sorokin, A. B.; Dutasta, J. P.; Martinez, A. *Chem. Commun.* **2013**, *49*, 1288–1290. doi:10.1039/C2CC37829A

(328) Smith, P. T.; Benke, B. P.; Cao, Z.; Kim, Y.; Nichols, E. M.; Kim, K.; Chang, C. J. *Angew. Chem. Int. Ed.* **2018**, *57*, 9684-9688. doi:10.1002/anie.201803873
(329) Feng, X.; Liao, P.; Jiang, J.; Shi, J.; Ke, Z.; Zhang, J. *ChemPhotoChem* **2019**, *3*, 1014-1019. doi:10.1002/cptc.201900058
(330) Koo, J.; Kim, I.; Kim, Y.; Cho, D.; Hwang, I. C.; Mukhopadhyay, R. D.; Song, H.; Ko, Y. H.; Dhamija, A.; Lee, H.; Hwang, W.; Kim, S.; Baik, M. H.; Kim, K. *Chem* **2020**, *6*. doi:10.1016/j.chempr.2020.10.002
(331) Liu, C.; Liu, K.; Wang, C.; Liu, H.; Wang, H.; Su, H.; Li, X.; Chen, B.; Jiang, J. *Nat. Commun.* **2020**, *11*, 1047. doi:10.1038/s41467-020-14831-x
(332) Yang, J.; Chatelet, B.; Dufaud, V.; Hérault, D.; Michaud-Chevallier, S.; Robert, V.; Dutasta, J.-P.; Martinez, A. *Angew. Chem. Int. Ed.* **2018**, *130*, 14408–14411. doi:10.1002/ANGE.201808291
(333) Chen, H. Y.; Gou, M.; Wang, J. B. *Chem. Commun.* **2017**, *53*, 3524–3526. doi:10.1039/C7CC00938K
(334) Chatelet, B.; Dufaud, V.; Dutasta, J. P.; Martinez, A. *J. Org. Chem.* **2014**, *79*, 8684–8688. doi:https://doi.org/10.1021/jo501457d
(335) Li, C.; Manick, A. D.; Jean, M.; Albalat, M.; Vanthuyne, N.; Dutasta, J. P.; Bugaut, X.; Chatelet, B.; Martinez, A. *J. Org. Chem.* **2021**, *86*, 15055–15062. doi:10.1021/ACS.JOC.1C01731
(336) Raytchev, P. D.; Martinez, A.; Gornitzka, H.; Dutasta, J. P. *J. Am. Chem. Soc.* **2011**, *133*, 2157–2159. doi:10.1021/JA1110333
(337) Hussain, W.; Giri, A.; Patra, A. *Sustain. Energy Fuels* **2019**, *3*, 2567–2571. doi:10.1039/c9se00394k
(338) Mukhtar, A.; Sarfaraz, S.; Ayub, K. *RSC Adv.* **2022**, *12*, 24397–24411. doi:10.1039/D2RA03399B
(339) Wang, H.; Jin, Y.; Sun, N.; Zhang, W.; Jiang, J. *Chem. Soc. Rev.* **2021**, *50*, 8874–8886. doi:10.1039/D0CS01142H
(340) Otte, M. *Eur. J. Org. Chem.* **2023**, *26*, e202300012. doi:10.1002/ejoc.202300012
(341) Vliegenthart, A. B.; Welling, F. A. L.; Roemelt, M.; Klein Gebbink, R. J. M.; Otte, M. *Org. Lett.* **2015**, *17*, 4172–4175. doi:10.1021/acs.orglett.5b01931
(342) Begato, F.; Licini, G.; Zonta, C. *Chem. Sci.* **2023**, *14*, 8147–8151. doi:10.1039/D3SC01368E
(343) Begato, F.; Penasa, R.; Licini, G.; Zonta, C. *ACS Sens.* **2022**, *7*, 1390–1394. doi:10.1021/acssensors.2c00038
(344) Bravin, C.; Licini, G.; Hunter, C. A.; Zonta, C. *Chem. Sci.* **2019**, *10*, 1466–1471. doi:10.1039/C8SC04406F
(345) Bravin, C.; Badetti, E.; Scaramuzzo, F. A.; Licini, G.; Zonta, C. *J. Am. Chem. Soc.* **2017**, *139*, 6456–6460. doi:10.1021/jacs.7b02341
(346) Yu, Y.; Rebek, J. *Acc. Chem. Res.* **2018**, *51*, 3031–3040. doi:10.1021/ACS.ACCOUNTS.8B00269
(347) Breslow, R.; Heyer, D. *Tetrahedron Lett.* **1983**, *24*, 5039–5042. doi:10.1016/S0040-4039(00)94035-8
(348) Breslow, R.; Zhang, X.; Huang, Y. *J. Am. Chem. Soc.* **1997**, *119*, 4535–4536. doi:10.1021/JA9704951
(349) Bogie, P. M.; Holloway, L. R.; Ngai, C.; Miller, T. F.; Grewal, D. K.; Hooley, R. J. *Chem. Eur. J.* **2019**, *25*, 10232–10238. doi:10.1002/chem.201902049
(350) Zhao, Z.; Fukushima, T.; Zharnikov, M. *J. Phys. Chem. C* **2023**, *127*, 15582–15590. doi:10.1021/ACS.JPCC.3C03083
(351) Ishiwari, F.; Shoji, Y.; Martin, C. J.; Fukushima, T. *Polym. J.* **2024**, *56*, 791–818. doi:10.1038/s41428-024-00920-x
(352) Yoon, I.; Suh, S. E.; Barros, S. A.; Chenoweth, D. M. *Org. Lett.* **2016**, *18*, 1096–1099. doi:10.1021/acs.orglett.6b00169
(353) Barros, S. A.; Yoon, I.; Suh, S. E.; Chenoweth, D. M. *Org. Lett.* **2016**, *18*, 2423–2426. doi:10.1021/acs.orglett.6b00945
(354) Woźny, M.; Mames, A.; Ratajczyk, T. *Molecules* **2022**, *27*, 250. doi:10.3390/MOLECULES27010250
(355) Ueno, K.; Nishii, Y.; Miura, M. *Org. Lett.* **2021**, *23*, 3552–3556. doi:10.1021/acs.orglett.1c00970
(356) Santolini, V.; Miklitz, M.; Berardo, E.; Jelfs, K. E. *Nanoscale* **2017**, *9*, 5280–5298. doi:10.1039/C7NR00703E

(357) Ono, K.; Ishikawa, T.; Masano, S.; Kawai, H.; Goto, K. *J. Am. Chem. Soc.* **2024**, *146*, 35. doi:10.1021/JACS.4C03798
(358) Zhang, G.; Mastalerz, M. *Chem. Soc. Rev.* **2014**, *43*, 1934–1947. doi:10.1039/C3CS60358J
(359) Schick, T. H. G.; Lauer, J. C.; Rominger, F.; Mastalerz, M. *Angew. Chem. Int. Ed.* **2019**, *58*, 1768–1773. doi:10.1002/anie.201814243
(360) Lauer, J. C.; Bhat, A. S.; Barwig, C.; Fritz, N.; Kirschbaum, T.; Rominger, F.; Mastalerz, M. *Chem. Eur. J.* **2022**, *28*, e202201527. doi:10.1002/chem.202201527
(361) Hu, X. Y.; Zhang, W.-S.; Rominger, F.; Wacker, I.; Schröder, R. R.; Mastalerz, M. *Chem. Commun.* **2017**, *53*, 8616–8619. doi:10.1039/c7cc03677a
(362) Bhat, A. S.; Elbert, S. M.; Zhang, W.-S.; Rominger, F.; Dieckmann, M.; Schröder, R. R.; Mastalerz, M. *Angew. Chem. Int. Ed.* **2019**, *58*, 8819–8823. doi:10.1002/anie.201903631
(363) Han, X.; Huang, J.; Yuan, C.; Liu, Y.; Cui, Y. *J. Am. Chem. Soc.* **2018**, *140*, 892–895. doi:10.1021/JACS.7B12110
(364) Waller, P. J.; Lyle, S. J.; Osborn Popp, T. M.; Diercks, C. S.; Reimer, J. A.; Yaghi, O. M. *J. Am. Chem. Soc.* **2016**, *138*, 15519–15522. doi:10.1021/jacs.6b08377
(365) Tateishi, T.; Yoshimura, M.; Tokuda, S.; Matsuda, F.; Fujita, D.; Furukawa, S. *Coord. Chem. Rev.* **2022**, *467*, 214612. doi:10.1016/J.CCR.2022.214612
(366) Berardo, E.; Greenaway, R. L.; Turcani, L.; Alston, B. M.; Bennison, M. J.; Miklitz, M.; Clowes, R.; Briggs, M. E.; Cooper, A. I.; Jelfs, K. E. *Nanoscale* **2018**, *10*, 22381–22388. doi:10.1039/c8nr06868b
(367) Dasary, H.; Jagan, R.; Chand, D. K. *Inorg. Chem.* **2018**, *57*, 12222–12231. doi:10.1021/acs.inorgchem.8b01884
(368) Sarkar, M.; Dasary, H.; Chand, D. K. *J. Organomet. Chem.* **2021**, *950*, 121984. doi:10.1016/j.jorganchem.2021.121984
(369) Mishra, S. S.; Kompella, S. V. K.; Krishnaswamy, S.; Balasubramanian, S.; Chand, D. K. *Inorg. Chem.* **2020**, *59*, 12884–12894. doi:10.1021/acs.inorgchem.0c01964
(370) Begato, F.; Licini, G.; Zonta, C. *Angew. Chem. Int. Ed.* **2023**, *62*, e202311153. doi:10.1002/ANIE.202311153
(371) Zhou, H.; Ao, Y. F.; Wang, D. X.; Wang, Q. Q. *J. Am. Chem. Soc.* **2022**, *144*, 16767–16772. doi:10.1021/JACS.2C08591
(372) Matsushima, T.; Kikkawa, S.; Azumaya, I.; Watanabe, S. *ChemistryOpen* **2018**, *7*, 278–281. doi:10.1002/OPEN.201800006
(373) Kumar, A.; Krishnaswamy, S.; Chand, D. K. *Angew. Chem. Int. Ed.* **2024**, e202416332. doi:10.1002/ANIE.202416332
(374) Ebbert, K. E.; Sendzik, F.; Neukirch, L.; Eberlein, L.; Platzek, A.; Kibies, P.; Kast, S. M.; Clever, G. H.; Ebbert, K. E.; Sendzik, F.; Neukirch, L.; Eberlein, L.; Platzek, A.; Kibies, P.; Kast, S. M.; Clever, G. H. *Angew. Chem. Int. Ed.* **2024**, e202416076. doi:10.1002/ANIE.202416076
(375) Johnson, A. M.; Hooley, R. J. *Inorg. Chem.* **2011**, *50*, 4671–4673. doi:10.1021/IC2001688
(376) Tarzia, A.; Jelfs, K. E. *Chem. Commun.* **2022**, *58*, 3717–3730. doi:10.1039/d2cc00532h
(377) Greenaway, R. L.; Jelfs, K. E. *Chempluschem* **2020**, *85*, 1813–1823. doi:10.1002/cplu.202000445
(378) Ollerton, K.; Greenaway, R. L.; Slater, A. G. *Front. Chem.* **2021**, *9*, 774987. doi:10.3389/fchem.2021.774987
(379) Greenaway, R. L.; Santolini, V.; Bennison, M. J.; Alston, B. M.; Pugh, C. J.; Little, M. A.; Miklitz, M.; Eden-Rump, E. G. B.; Clowes, R.; Shakil, A.; Cuthbertson, H. J.; Armstrong, H.; Briggs, M. E.; Jelfs, K. E.; Cooper, A. I. *Nat. Commun.* **2018**, *9*, 2849. doi:10.1038/s41467-018-05271-9
(380) Jelfs, K. E. Chapter 1. Introduction to Computational Modelling of Microporous Materials. In *Computer Simulation of Porous Materials: Current Approaches and Future Opportunities*; Jelfs, K. E., Ed.; The Royal Society of Chemistry, 2021; pp 1–26. doi:10.1039/9781839163319-00001
(381) Turcani, L.; Berardo, E.; Jelfs, K. E. *J. Comput. Chem.* **2018**, *39*, 1931–1942. doi:10.1002/JCC.25377
(382) Yuan, Q.; Szczypiński, F. T.; Jelfs, K. E. *Digital Discovery* **2022**, *1*, 127–138. doi:10.1039/D1DD00039J
(383) Berardo, E.; Turcani, L.; Miklitz, M.; Jelfs, K. E. *Chem. Sci.* **2018**, *9*, 8513–8527. doi:10.1039/c8sc03560a
(384) Holden, D.; Jelfs, K. E.; Cooper, A. I.; Trewin, A.; Willock, D. J. *J. Phys. Chem. C* **2012**, *116*, 16639–16651. doi:10.1021/JP305129W

(385) Greenaway, R. L.; Santolini, V.; Pulido, A.; Little, M. A.; Alston, B. M.; Briggs, M. E.; Day, G. M.; Cooper, A. I.; Jelfs, K. E. *Angew. Chem. Int. Ed.* **2019**, *58*, 16275–16281. doi:10.1002/anie.201909237
(386) Piskorz, T. K.; Martí-Centelles, V.; Young, T. A.; Lusby, P. J.; Duarte, F. *ACS Catal.* **2022**, 5806–5826. doi:https://doi.org/10.1021/acscatal.2c00837
(387) Piskorz, T. K.; Lee, B.; Zhan, S.; Duarte, F. *J. Chem. Theory Comput.* **2024**, *20*, 9060–9071. doi:10.1021/ACS.JCTC.4C00850
(388) Chen, K.; Arnold, F. H. *Nat. Catal.* **2020**, *3*, 203–213. doi:10.1038/s41929-019-0385-5
(389) Lovelock, S. L.; Crawshaw, R.; Basler, S.; Levy, C.; Baker, D.; Hilvert, D.; Green, A. P. *Nature* **2022**, *606*, 49–58. doi:10.1038/s41586-022-04456-z
(390) Leveson-Gower, R. B.; Mayer, C.; Roelfes, G. *Nat. Rev. Chem.* **2019**, *3*, 687–705. doi:10.1038/s41570-019-0143-x
(391) Longwitz, L.; Leveson-Gower, R. B.; Rozeboom, H. J.; Thunnissen, A. M. W. H.; Roelfes, G. *Nature* **2024**, *629*, 824–829. doi:10.1038/s41586-024-07391-3
(392) Richard, J. P. *J. Am. Chem. Soc.* **2019**, *141*, 3320–3331. doi:10.1021/JACS.8B10836
(393) Huber, R. *Biochem. Soc. Trans.* **1987**, *15*, 1009–1020. doi:10.1042/BST0151009
(394) Karshikoff, A.; Nilsson, L.; Ladenstein, R. *FEBS J.* **2015**, *282*, 3899–3917. doi:10.1111/FEBS.13343
(395) Ma, B.; Nussinov, R. *Curr. Opin. Chem. Biol.* **2010**, *14*, 652–659. doi:10.1016/J.CBPA.2010.08.012
(396) Kremer, C.; Lützen, A. *Chem. Eur. J.* **2013**, *19*, 6162–6196. doi:10.1002/CHEM.201203814
(397) Masar, M. S.; Gianneschi, N. C.; Oliveri, C. G.; Stern, C. L.; Nguyen, S. B. T.; Mirkin, C. A. *J. Am. Chem. Soc.* **2007**, *129*, 10149–10158. doi:10.1021/JA0711516
(398) Gallagher, J. M.; Roberts, B. M. W.; Borsley, S.; Leigh, D. A. *Chem* **2024**, *10*, 855–866. doi:10.1016/j.chempr.2023.10.019
(399) Otten, R.; Pádua, R. A. P.; Bunze, H. A.; Nguyen, V.; Pitsawong, W.; Patterson, M.; Sui, S.; Perry, S. L.; Cohen, A. E.; Hilvert, D.; Kern, D. *Science* **2020**, *370*, 1442–1446. doi:10.1126/SCIENCE.ABD3623
(400) Nagel, Z. D.; Klinman, J. P. *Nat. Chem. Bio.* **2009**, *5*, 543–550. doi:10.1038/nchembio.204
(401) Eisenmesser, E. Z.; Bosco, D. A.; Akke, M.; Kern, D. *Science* **2002**, *295*, 1520–1523. doi:10.1126/SCIENCE.1066176

(402) Henzler-Wildman, K.; Kern, D. *Nature* **2007**, *450*, 964–972. doi:10.1038/Nature06522
(403) Molenveld, P.; Engbersen, J. F. J.; Reinhoudt, D. N. *Chem. Soc. Rev.* **2000**, *29*, 75–86. doi:10.1039/A804295K
(404) Cacciapaglia, R.; Di Stefano, S.; Mandolini, L.; Salvio, R. *Supramol. Chem.* **2013**, *25*, 537–554. doi:10.1080/10610278.2013.824578
(405) Mroz, A. M.; Posligua, V.; Tarzia, A.; Wolpert, E. H.; Jelfs, K. E. *J. Am. Chem. Soc.* **2022**, *144*, 18730–18743. doi:10.1021/JACS.2C06833
(406) Zhu, Q.; Qu, H.; Avci, G.; Hafizi, R.; Zhao, C.; Day, G. M.; Jelfs, K. E.; Little, M. A.; Cooper, A. I. *Nat. Synth.* **2024**, *3*, 825–834. doi:10.1038/s44160-024-00531-7
(407) Liu, Y.; Zhu, G.; You, W.; Tang, H.; Jones, C. W.; Lively, R. P.; Sholl, D. S. *J. Phys. Chem. C* **2019**, *123*, 1720–1729. doi:10.1021/ACS.JPCC.8B08838
(408) Bernabei, M.; Pérez-Soto, R.; Gómez García, I.; Haranczyk, M. *Mol. Syst. Des. Eng.* **2018**, *3*, 942–950. doi:10.1039/C8ME00055G
(409) Basford, A. R.; Bennett, S. K.; Xiao, M.; Turcani, L.; Allen, J.; Jelfs, K. E.; Greenaway, R. L. *Chem. Sci.* **2024**, *15*, 6331–6348. doi:10.1039/D3SC06133G
(410) Tarzia, A.; Wolpert, E. H.; Jelfs, K. E.; Pavan, G. M. *Chem. Sci.* **2023**, *14*, 12506–12517. doi:10.1039/D3SC03991A
(411) Nakajima, K.; Fujiwara, K.; Pan, W.; Okuda, H. *Nat. Mat.* **2004**, *3*, 838–838. doi:10.1038/nmat1282x
(412) Corbella, M.; Pinto, G. P.; Kamerlin, S. C. L. *Nat. Chem. Rev.* **2023**, *7*, 536–547. doi:10.1038/s41570-023-00495-w
(413) Young, T. A.; Gheorghe, R.; Duarte, F. *J. Chem. Inf. Model* **2020**, *60*, 3546–3557. doi:10.1021/ACS.JCIM.0C00519
(414) Young, T. A.; Silcock, J. J.; Sterling, A. J.; Duarte, F. *Angew. Chem. Int. Ed.* **2021**, *60*, 4266–4274. doi:10.1002/ANIE.202011941

(415) Vaissier Welborn, V.; Head-Gordon, T. *J. Phys. Chem. Lett.* **2018**, *9*, 3814–3818. doi:10.1021/ACS.JPCLETT.8B01710
(416) Vaissier Welborn, V.; Head-Gordon, T. *Chem. Rev.* **2019**, *119*, 6613–6630. doi:10.1021/acs.chemrev.8b00399
(417) Welborn, V. V.; Ruiz Pestana, L.; Head-Gordon, T. *Nat. Catal.* **2018**, *1*, 649–655. doi:10.1038/s41929-018-0109-2
(418) Dai, T.; Vijayakrishnan, S.; Szczypiński, F. T.; Ayme, J. F.; Simaei, E.; Fellowes, T.; Clowes, R.; Kotopanov, L.; Shields, C. E.; Zhou, Z.; Ward, J. W.; Cooper, A. I. *Nature* **2024**, *635*, 890–897. doi:10.1038/s41586-024-08173-7
(419) Salvio, R.; D'Abramo, M. *Eur. J. Org. Chem.* **2020**, *2020*, 6004–6011. doi:10.1002/EJOC.202001022
(420) Webb, T. H.; Wilcox, C. S. *Chem. Soc. Rev.* **1993**, *22*, 383. doi:10.1039/cs9932200383
(421) Wylie, R. S.; Sanders, J. K. M. *Tetrahedron* **1995**, *51*, 513–526. doi:10.1016/0040-4020(94)00912-E
(422) Gramage-Doria, R.; Hessels, J.; Leenders, S. H. A. M.; Tröppner, O.; Dürr, M.; Ivanovic, I.; Reek, J. N. H. *Angew. Chem. Int. Ed.* **2014**, *53*, 13380–13384. doi:10.1002/ANIE.201406415
(423) O'Sullivan, M. C.; Sprafke, J. K.; Kondratuk, D. V.; Rinfray, C.; Claridge, T. D. W.; Saywell, A.; Blunt, M. O.; O'Shea, J. N.; Beton, P. H.; Malfois, M.; Anderson, H. L. *Nature* **2011**, *469*, 72–75. doi:10.1038/Nature09683
(424) Gotfredsen, H.; Deng, J.-R.; Van Raden, J. M.; Righetto, M.; Hergenhahn, J.; Clarke, M.; Bellamy-Carter, A.; Hart, J.; O'Shea, J.; Claridge, T. D. W.; Duarte, F.; Saywell, A.; Herz, L. M.; Anderson, H. L. *Nat. Chem.* **2022**, 14, 1436–1442. doi:10.1038/s41557-022-01032-w
(425) Mecozzi, S.; Rebek, Jr., J. *Chem. Eur. J.* **1998**, *4*, 1016–1022. doi:10.1002/(SICI)1521-3765(19980615)4:6<1016::AID-CHEM1016>3.0.CO;2-B
(426) Plante, O. J.; Palmacci, E. R.; Seeberger, P. H. *Science* **2001**, *291*, 1523–1527. doi:10.1126/SCIENCE.1057324
(427) Gorelik, D. J.; Desai, S. P.; Jdanova, S.; Turner, J. A.; Taylor, M. S. *Chem. Sci.* **2024**, *15*, 1204–1236. doi:10.1039/D3SC05400D
(428) Blaszczyk, S. A.; Homan, T. C.; Tang, W. *Carbohydr. Res.* **2019**, *471*, 64–77. doi:10.1016/J.CARRES.2018.11.012
(429) Ghosal, S.; Das, A.; Roy, D.; Dasgupta, J. *Nat. Commun.* **2024**, *15*, 1810. doi:10.1038/s41467-024-45991-9
(430) Pullen, S.; Löffler, S.; Platzek, A.; Holstein, J. J.; Clever, G. H. *Dalton Trans.* **2020**, *49*, 9404–9410. doi:10.1039/D0DT01674H
(431) Großkopf, J.; Kratz, T.; Rigotti, T.; Bach, T. *Chem. Rev.* **2022**, *122*, 1626–1653. doi:10.1021/ACS.CHEMREV.1C00272
(432) Lee, H. S.; Jee, S.; Kim, R.; Bui, H. T.; Kim, B.; Kim, J. K.; Park, K. S.; Choi, W.; Kim, W.; Choi, K. M. *Energy Environ. Sci.* **2020**, *13*, 519–526. doi:10.1039/C9EE02619C
(433) Das, A.; Mandal, I.; Venkatramani, R.; Dasgupta, J. *Sci. Adv.* **2019**, *5*, eaav4806. doi:10.1126/SCIADV.AAV4806
(434) Spicer, R. L.; O'Connor, H. M.; Ben-Tal, Y.; Zhou, H.; Boaler, P. J.; Milne, F. C.; Brechin, E. K.; Lloyd-Jones, G. C.; Lusby, P. J. *Chem. Sci.* **2023**, *14*, 14140–14145. doi:10.1039/D3SC04877B
(435) Merlau, M. L.; Grande, W. J.; Nguyen, S. T.; Hupp, J. T. *J. Mol. Catal. A Chem.* **2000**, *156*, 79–84. doi:10.1016/S1381-1169(99)00399-4
(436) Kennedy, A. D. W.; DiNardi, R. G.; Fillbrook, L. L.; Donald, W. A.; Beves, J. E. *Chem. Eur. J.* **2022**, *28*, e202104461. doi:10.1002/CHEM.202104461
(437) DiNardi, R. G.; Douglas, A. O.; Tian, R.; Price, J. R.; Tajik, M.; Donald, W. A.; Beves, J. E. *Angew. Chem. Int. Ed.* **2022**, *61*, e202205701. doi:10.1002/ANIE.202205701
(438) Shaik, S. *ACS Physical Chemistry Au* **2024**, *4*, 191–201. doi:10.1021/ACSPHYSCHEMAU.3C00064
(439) Siddiqui, S. A.; Stuyver, T.; Shaik, S.; Dubey, K. D. *JACS Au* **2023**, *3*, 3259–3269. doi:10.1021/JACSAU.3C00536
(440) Léonard, N. G.; Dhaoui, R.; Chantarojsiri, T.; Yang, J. Y. *ACS Catal* **2021**, *11*, 10923–10932. doi:10.1021/ACSCATAL.1C02084
(441) Petrovic, D.; Risso, V. A.; Kamerlin, S. C. L.; Sanchez-Ruiz, J. M. *J. R. Soc. Interface* **2018**, *15*, 20180330. doi:10.1098/rsif.2018.0330
